# Sperm bauplan and function and underlying processes of sperm formation and selection

**DOI:** 10.1152/physrev.00009.2020

**Published:** 2021-04-21

**Authors:** Maria Eugenia Teves, Eduardo R. S. Roldan

**Affiliations:** ^1^Department of Obstetrics and Gynecology, Virginia Commonwealth University, Richmond, Virginia; ^2^Department of Biodiversity and Evolutionary Biology, Museo Nacional de Ciencias Naturales (CSIC), Madrid, Spain

**Keywords:** male fertility, spermatogenesis, spermatozoa, sperm form, sperm function

## Abstract

The spermatozoon is a highly differentiated and polarized cell, with two main structures: the head, containing a haploid nucleus and the acrosomal exocytotic granule, and the flagellum, which generates energy and propels the cell; both structures are connected by the neck. The sperm’s main aim is to participate in fertilization, thus activating development. Despite this common bauplan and function, there is an enormous diversity in structure and performance of sperm cells. For example, mammalian spermatozoa may exhibit several head patterns and overall sperm lengths ranging from ∼30 to 350 µm. Mechanisms of transport in the female tract, preparation for fertilization, and recognition of and interaction with the oocyte also show considerable variation. There has been much interest in understanding the origin of this diversity, both in evolutionary terms and in relation to mechanisms underlying sperm differentiation in the testis. Here, relationships between sperm bauplan and function are examined at two levels: first, by analyzing the selective forces that drive changes in sperm structure and physiology to understand the adaptive values of this variation and impact on male reproductive success and second, by examining cellular and molecular mechanisms of sperm formation in the testis that may explain how differentiation can give rise to such a wide array of sperm forms and functions.

## Graphical abstract

**Figure:**
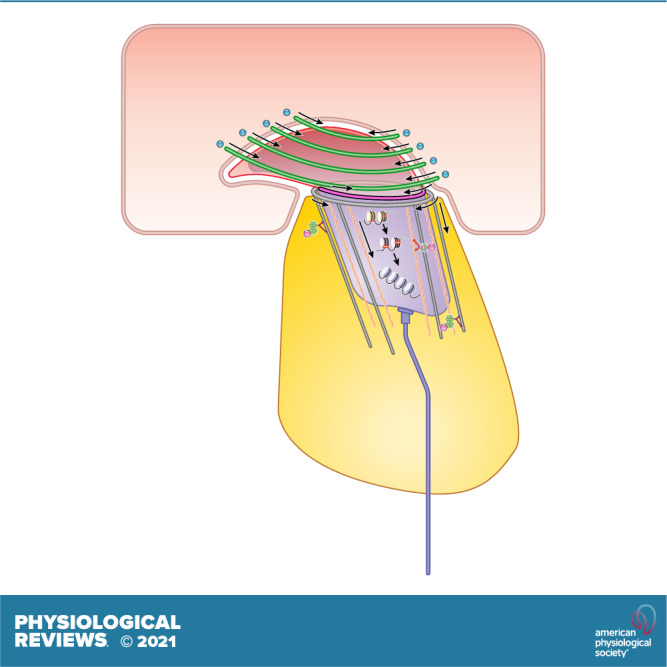

## 1. INTRODUCTION

The sperm cell is a highly polarized and differentiated cell, whose main goal is to reach the oocyte and participate in fertilization (1). To do so, it must embark on a long journey, and success in this endeavor relies on structurally and functionally sound cells. Despite this common task, sperm cells from different species vary in structure and mechanisms underlying their functions. To understand how and why such variation has originated, it is possible to inquire about the processes of sperm formation (2–4) and about the evolutionary forces that may operate in selecting spermatozoa (5–8). There has been a long-standing interest in both, but progress has been slow, particularly when trying to integrate them. In this review we summarize aspects of sperm structure and function and attempt to relate them to processes of spermatogenesis and postcopulatory sexual selection. Our general aim is to present this outline with a view toward a better understanding of sperm biology and, in addition, to provide background for assessments of sperm fertilizing capacity and for the development of improved methods of assisted reproduction.

CLINICAL HIGHLIGHTS

1. Sperm morphology is closely associated to sperm function. In humans, sperm morphology varies considerably even within the same individual. This pleomorphic characteristic of spermatozoa has led to problems when devising methods to assessing them in a clinical context or when evaluating the effect of genetic or environmental stresses. Very strict criteria for the evaluation of human sperm morphology, such as those recommended by the World Health Organization, result in a threshold for normality of only ∼4% of spermatozoa.
2. Sperm variation raises several questions, such as what normal sperm cells are, how we can identify them, how we can overcome the difficulty of guaranteeing standards across laboratories, and whether we can design new approaches for morphology assessments in a clinical context.
3. Because sperm form is closely related to its function, abnormal sperm may not swim adequately and reach the site of fertilization or fertilize in vitro. This not only relates to hydrodynamic efficiency of swimming but is also indicative of problems during sperm formation that may influence function at other levels (signaling, bioenergetics) or be a sign of sperm DNA damage. The latter is relevant because mutations in the father could lead to genetic diseases in offspring.
4. Altogether, a better knowledge of processes of sperm formation, and selection methods that rely on form and function, may lead to better diagnosis and improved techniques to assist (or control) human reproduction.

Overall, sperm structure is highly conserved throughout animals, although exceptions do exist. In general terms, the sperm bauplan (i.e., its design) consists of a head and a flagellum (or tail) (9–11). The sperm head has the nucleus and an exocytotic granule called the acrosome. A neck, or connecting piece, attaches the sperm head to the flagellum and contains typical or atypical centrioles (12). The flagellum consists of the midpiece and the principal and end pieces and usually has the machinery to produce energy in the form of ATP. It also provides the propulsive force for swimming (9–11).

Efficiency in sperm function relates to several aspects. First, the sperm head carrying the nucleus is streamlined for hydrodynamic efficiency. Second, the nucleus is reduced in size by compaction of chromatin, which occurs via histone replacement and protamine binding to DNA. Third, chromatin compaction also protects DNA integrity, thus minimizing damage from intra- and extracellular stress. Fourth, because genes are silenced by protamine action and chromatin compaction, the sperm has limited repair capacity. Fifth, remodeling of the sperm head takes place together with the development of a flagellum for cell motion, which requires integration of formation of both components. Sixth, sperm movement relies on propulsive forces generated by the flagellum and energy production. Finally, once the sperm cells are released or transferred to the female tract, a series of additional structural and functional changes take place in preparation for fertilization.

The study of model organisms from a few taxa to understand reproductive physiology is advantageous for identification of common themes in structure and function. However, this occasionally has led us to forget that these models may represent just some limited examples in comparison to the diversity that exists in nature. Paying attention to such diversity may be informative as to the role played by structural components and for a better understanding of molecular mechanisms that control cell formation and function. To understand the diversity of sperm cells, with regard to both structure and their physiology, it is important to bear in mind that sperm cells are manufactured under delicate genetic control but that this process is also susceptible to environmental factors. After sperm production in the testes, sperm cells undergo further processing in the male and female tracts, which are also under genetic control and may be modified by environmental conditions.

Mechanisms of sperm formation in the testis are generally conserved in vertebrates, with a finely orchestrated interaction between somatic and germ cells in the seminiferous tubules and a series of phases that involve proliferation, meiosis, and differentiation (13–15), but, given that there is considerable diversity in the structure of the sperm cell, it is predictable that such diversity could be explained by differences in both the architecture of the testis and the kinetics of sperm proliferation or differentiation. This will be particularly relevant in the final stages of spermatogenesis, when the sperm cell is formed and when considerable remodeling of the nucleus and the generation of sperm-specific organelles and structures take place. It is thus not surprising that much variation exists in the underlying cellular and molecular mechanisms regulating this last step of spermatogenesis, and this could be one source of heterogeneity in sperm cells.

Several selective forces may be responsible for the evolution of the known diversity in sperm structure, together with variation in cell functions (e.g., the provision of energy, propulsion, exocytosis of a single acrosomal granule, interaction with the female gamete, and penetration of the oocyte vestments). In vertebrates, fertilization could be either external or internal, and this would affect the sources of energy that the sperm may use to sustain propulsion. In addition, fertilization mode defines different environments (regarding both space and time) in which the sperm needs to survive in order to encounter an oocyte and engage in fertilization. The evolution of internal fertilization may have heavily influenced the structure and the function of the male gamete (16), considering that the sperm cell has to interact with the female tract and, in addition, undergo additional steps to be able to interact with the oocyte. The female tract may present a series of formidable barriers that the sperm has to overcome, and this could serve as the basis for female selection. Furthermore, females may mate with more than one male, and under these circumstances sperm from rival males will have to compete in order to achieve fertilizations. The latter scenarios are the basis for postcopulatory sexual selection, which may have heavily influenced the evolution of sperm shape, size, and function.

There have been several recent reviews covering some of the issues mentioned above; they are referred to in the relevant sections. We have also recently reviewed some information on sperm function or sperm formation and on evolutionary processes in spermatozoa. Knowledge on protein/vesicle transport during spermatogenesis has received particular attention, and we have presented an in silico analysis that identified, for the first time, the molecular interactome of proteins involved in protein trafficking during spermiogenesis (17). This overview has identified gaps in knowledge and challenges for future research. We have recently addressed several levels of proximate and evolutionary explanations that can be used as framework to gain integrated knowledge of sperm biology, particularly structure and function of sperm cells attending fertilization (18), so these issues are not covered in detail here. To assess diversity in sperm morphology and function, it is necessary to resort to a series of methods for image analysis and cellular and molecular functional tests that can inform on sperm performance; various approaches for such sperm assessments have been reviewed recently (19) and the implications for male fertility considered in this context (20). Finally, the principles of sperm competition have been examined in connection to the development of new tests for the analysis of sperm morphology or sperm performance (21).

In the present overview, we focus on two particular levels of analysis. On the one hand, we address evolutionary forces (postcopulatory sexual selection) in relation to sperm structure and spermatogenesis. On the other hand, we deal with proximate (mechanistic) explanations of molecular changes underlying spermiogenesis, with a particular interest in structural diversity and the origin of biological novelties in sperm. The rationale for this review is thus to integrate various aspects of sperm biology in such a way that it could be appealing to students and specialists interested in cellular and molecular aspects of sperm formation, function, and fertility and also those interested in understanding evolutionary issues of sperm biology. In addition, we anticipate that it may provide background for those focusing on clinical aspects of male reproduction or in areas connected to animal production. This review therefore covers the following. First, it gives an overview of the sperm bauplan, that is, the structure of the sperm cell and its main components. The attention is mostly on the shape and the size of the sperm head, because considerable recent research exists on these topics. We also present an overview of sperm head diversity in mammals, with special attention to rodents, a group of species in which a series of novel structures have evolved. Second, we examine the possible role of selective forces on changes in the sperm bauplan and function. Third, we review a series of cellular and molecular mechanisms that remodel and reshape the male germ cell in the stages after meiosis. In this phase (spermiogenesis), the round spermatid, which has a structure not very different from a round somatic cell (except for the fact that it is a haploid cell), changes through different steps into a highly differentiated and compartmentalized cell. From a shape that is somewhat similar among species, it develops into an array of very different shapes and sizes. Our goal therefore is to illustrate evolution of form and function in a highly specialized cell and summarize mechanisms that can explain the onset of biological novelty and diversity in relation to selective forces and processes of cell formation. Finally, we aim to raise awareness of areas that need further research and that could benefit from using a comparative and evolutionary approach to understand diversity in form and function and the underlying molecular mechanisms.

## 2. SPERM STRUCTURE AND FUNCTION

### 2.1. Sperm Shape and Size

#### 2.1.1. Overall structure.

The general morphology of an organism or a cell constitutes its bauplan. A bauplan (which can also be regarded as a design or blueprint) is the general configuration, the structure and organization of a body plan. However, although the term makes reference to a design, it does not mean that it is entirely determined by a genetic code, because there are a number of factors that can influence its development, both within the organism and in the environment. Bauplan thus refers to the morphology that, in turn, corresponds to the shape (form) and size of an organism or a cell. Size, in turn, relates to both absolute and relative dimensions of its different parts. Finally, ultrastructure and subcellular components relating to the organization and distribution of cell organelles are also relevant.

**FIGURE 1.**
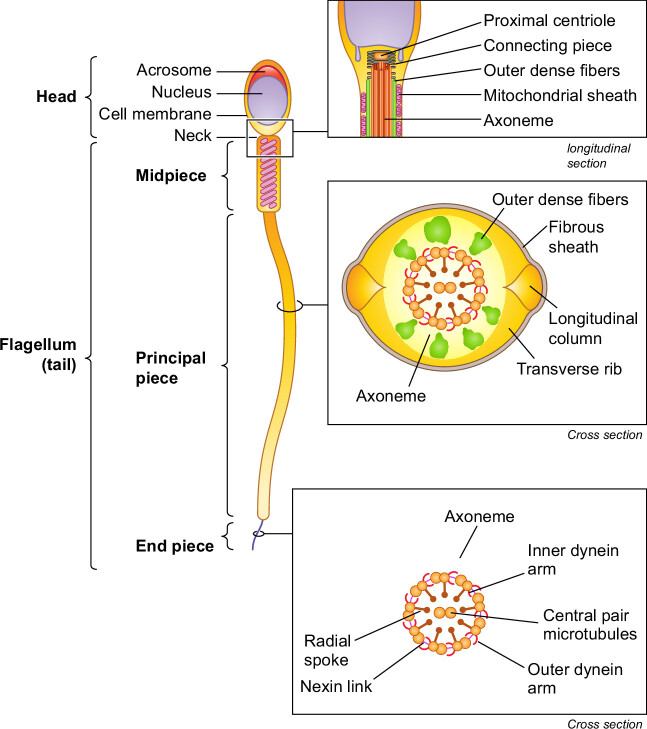
Sperm structure and compartments. General structure of spermatozoa, which consists of a head and a flagellum. The head contains the nucleus and the acrosomal granule. The flagellum, which is connected to the head by the neck, is divided into 3 components: the midpiece with the mitochondria, the principal piece, and the end piece. The axial filament contains the 9 + 2 array of the microtubules. This sperm pattern shows much variation across vertebrate taxa.

In general, the bauplan of sperm cells is conserved in animals. What follows is a summary of the general structure of sperm cells. Several comprehensive reviews with detailed information on sperm structure can be found in the recent literature (9–12, 22, 23). Spermatozoa have two major compartments, the head and the flagellum (or tail) (FIGURE 1). The sperm head contains the haploid nucleus, with highly compacted chromatin that carries hereditary information in the DNA, and the acrosome, an exocytotic granule with enzymes that upon release help the sperm cells penetrate the oocyte vestments during fertilization. Extranuclear and extra-acrosomal regions can be identified in the sperm head. Furthermore, there are cytoplasmic compartments defined by acrosomal membranes, the nuclear envelope, and plasma membrane. The perinuclear theca is a sheath that encases the nucleus. The perforatorium is a specialized structure found in the apical region of the subacrosomal perinuclear theca that serves to anchor the acrosome to the anterior region of the nucleus.

The flagellum attaches to the sperm head via the neck (or connecting piece), which includes typical or atypical proximal and distal centrioles. The flagellum has three recognizable regions: the midpiece, the principal piece, and the terminal piece. The midpiece has the neck as the anterior limit and the annulus, which is a transverse ring of dense material, as the posterior limit. The midpiece contains mitochondria that generate ATP via oxidative phosphorylation, whereas the principal piece can generate ATP via glycolysis. The energy from the ATP is used by the principal piece to provide the propulsive force through a structure called the axial filament or axoneme. The axoneme extends usually from the neck to the end of the flagellum and has a distinctive 9 + 2 microtubule structure in most spermatozoa. Proteins associated with the axonemal microtubules hydrolyze the ATP and convert the chemical energy released from the ATP into mechanical forces that slide the microtubules and generate the flagellar motility. This propulsion and pattern of flagellar movement is typical for the different species (24–27).

Overall, spermatozoa are highly polarized and extremely differentiated cells. Importantly, their repair capacity is very limited, thus being susceptible to various factors that can generate irreversible damage. The sperm bauplan, with many of its peculiarities, thus relates to the need to protect the cell from this potential damage and carry the genetic load to the oocyte.

Despite their common structure, spermatozoa from some species depart from this general bauplan. In some species sperm lack a flagellum or may bear two or more. In others, the acrosome may be absent, as in many fish species. And yet, in others, spermatozoa may bear no genome and may act to carry other sperm cells to the site of fertilization.

#### 2.1.2. Diversity.

**FIGURE 2.**
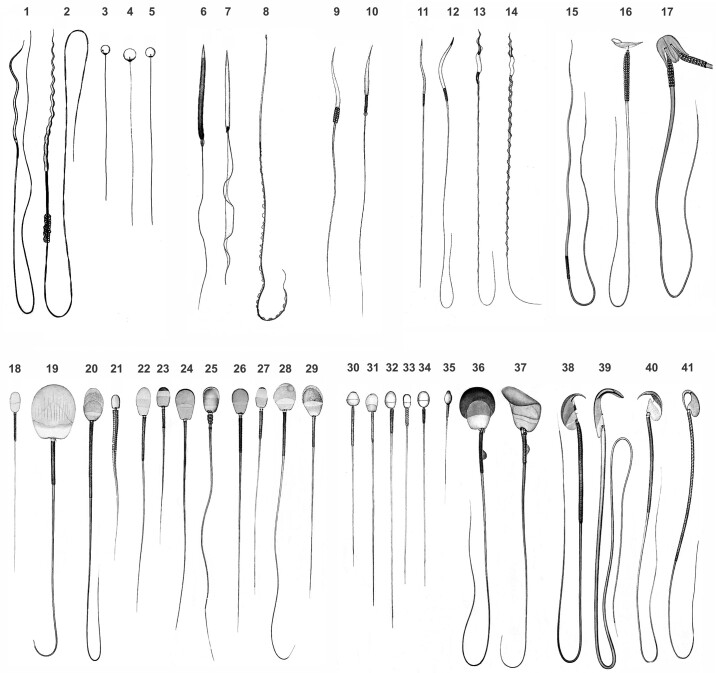
Sperm diversity in vertebrates. Illustrations are drawings from Gustav Retzius (29), and they are presumably all at the same magnification and scale (30). For each species, information regarding the source of each drawing is given in parentheses, and it includes volume number (NF), plate number (Roman numbers), and figure number within each plate. Names of species follow those given by Retzius, and current names are given in parentheses when appropriate. Fishes, Chondrichthyes: (**1**) *Chimaera* sp. (NF14 XXVII-1); (**2**) *Squalus acanthias* (NF14 XXVIII-16); Osteichthyes: (**3**) *Esox* sp., freshwater species (NF12 XIX-9); (**4**) *Salmo* sp., fresh and seawater species (NF12 XX-1); (**5**) *Pleuronectes* sp., seawater (NF12 XX-16). Amphibians, Anurans: (**6**) *Hyla arborea* (NF13 XXVII-1); (**7**) *Bufo vulgaris* (= *Bufo bufo*) (NF13 XXIV-1); Urodeles: **(8**) *Salamandra maculosa* (= *Salamandra salamandra*) (NF13 XX-1). Reptiles, Chelonia: (**9**) *Testudo* sp. (NF13 XXVIII-15); Squamata: (**10**) *Platydactylus* sp. (= *Rhacodactylus* sp.) (NF13 XXVIII-6). Birds, nonpasserines: (**11**) *Struthio* sp. (NF16 XXV-20); (**12**) *Gallus* sp. (NF14 XXXI-1); passerines: (**13**) *Passer passer* (NF14 XXXV-1); (**14)** *Sturnus* sp. (NF14 XXXVI-17). Mammals, monotremes: (**15**) *Tachyglossus* sp. (NF13 XXIX-1); marsupials: (**16**) *Petrogale* sp. (NF13 XXXI-9); (**17**) *Didelphys* sp. showing paired spermatozoa (NF14 XXXVIII-1); Eutheria, Afrotheria: (**18**) *Elephas* sp. (NF16 XXV-1); Xenarthra: (**19**) *Dasypus villosus* (= *Chaetophractus villosus*) (NF13 XXXII-1); Insectivora: (**20**) *Talpa* sp. (NF14 XXXIX-1); Chiroptera: (**21**) *Vesperugo pipistrellus* (= *Pipistrellus pipistrellus*) (NF13 XXIX-3); Cetartiodactyla: (**22**) *Equus* sp. (NF14 L-1); (**23**) *Dicotyles* sp. (NF14 XLIX-1); (**24**) *Cervus elaphus* (NF14 LI-1); (**25**) *Globicephalus* sp. (= *Globicephala* sp.) (NF14 LIV-1); Carnivora: (**26**) *Canis familiaris* (NF14 LVI-1); (**27**) *Felis catus* (NF14 LVI-20); (**28**) *Meles* sp. (NF14 LVII-13); (**29**) *Halichaerus* sp. (NF14 LVII-28); Primates, prosimians: (**30**) *Lemur* sp. (NF17 XVI-1); New World monkeys: (**31**) *Chrysothrix sciurea* (= *Saimiri sciureus*) (NF19 XXI-1); Old World mondeys: (**32**) *Innuus* sp. (= *Macaca sylvanu*s) (NF17 XVI-1); great apes: (**33**) *Gorilla* sp. (NF17 XVI-27); (**34**) *Homo* sp. (NF17 XVI-35); Rodentia, Histrocoidea: (**35**) *Hystrix* sp. (NF14 XL-28); caviomorphs: (**36**) *Cavia* sp. (NF14 XLV-1); Sciuromorpha: (**37**) *Sciurus vulgaris* (NF14 XLI-1); Myomorpha: (**38**) *Mus musculus* (NF14 XLVI-1); (**39**) *Mus norvegicus* (= *Rattus norvegicus*) (NF14 XLVIII-1); (**40**) *Microtus terrestris* (= *Microtus arvalis*) (NF14 XLVI-1); (**41**) *Lemmus* sp. (NF14 XLV-15).

It has been known for a long time that spermatozoa have an extraordinary diversity in morphology, with interspecific differences being greater than those seen for any other cell type (28) (FIGURE 2). This diversity has puzzled scientists for a long time, and the reasons for this variation have been difficult to grasp, to the extreme that it has, at some point, been considered to have no adaptive value (31). This diversity in spermatozoa occurs in agreement with diversity in reproductive patterns, and the possibility exists that changes in reproductive strategies may have influenced sperm structure and function. In vertebrates, for example, taxa vary in the mode of fertilization (which could be external or internal), specialized oocyte structures (32), social structures and mating behaviors, and processes of postcopulatory sexual selection (5), and these characteristics could have influenced sperm evolution. Below, a summary is presented of the main aspects regarding sperm morphology in vertebrates. This is necessarily a general overview because information is lacking for many taxa. In any case, what we already know about sperm morphology in this diverse group of animals underscores the huge diversity in shape and size (FIGURE 2). Information on sperm attributes for the main vertebrate taxa is summarized in TABLE 1.

**Table 1.** Diversity of sperm traits in vertebrates

Class	Sub-/Infraclasses	Sperm Traits
Fishes	Chondrichthyes (sharks, skates, rays, chimeras)	Head is helical and long (>30 µm). Acrosome is conical and moderately elongated. Axoneme with 9 + 2 or 9 + 0 microtubular arrangement. Flagellum contains longitudinal columns and a midpiece with mitochondria. Sperm length: 75–230 µm.
	Osteichthyes (bony fish)	Elongated nucleus. Midpiece is short with 2 centrioles, mitochondria, and dense body. 9 + 0 or 9 + 2 axoneme. Flagellum has membrane with 2 lateral extensions (side fins) arranged in line with central axonemal tubules. Teleost sperm have elongated midpiece and no acrosome. Sperm length: 13–272 µm, with 1 outlier (*N. forsteri*).
Amphibians	Anurans (frogs and toads)	*Primitive anurans:* Nucleus with poorly compacted chromatin; endonuclear canal; perforatorium not well defined; acrosome is conical. Short axial rod surrounded by mitochondria. Flagellum insertion is postnuclear in most primitive taxa. *Higher anurans:* No perforatorium, endonuclear canal and axial rod. Sperm cell length: 19–240 µm.
	Urodeles (salamanders)	Highly compacted chromatin, nuclear ridge, discrete endonuclear canal, perforatorium, clover-shaped acrosome with prominent acrosomal barb and subacrosomal rod. Long and stiff axial rod with abundant encircling mitochondria. Sperm length: ∼170–880 µm.
	Caecilians (Gymnophiona)	Sperm resemble those of urodeles. Acrosome exhibits 3 zones and a base plate. Axoneme and axial rod are in proximity in the midpiece. Tail has undulating membrane. Sperm length: ∼60–255 µm.
Reptiles	Chelonia (turtles, tortoises)	Compact nucleus with deeply penetrating intranuclear tubes and perforatorial cap. Peculiar distal centriole with central microtubules along entire caudal length, surrounded by 9 peripheral triplets. Laminated mitochondria. Sperm length: ∼50–55 µm.
	Rhynchocephalia (tuatara)	Long helical nucleus with a tip constriction and paired endonuclear canals; conical acrosome. Spheroidal mitochondria with concentric cristae and dense lateral body. Annulus; long principal piece with dense fibrous sheath; short end piece. Sperm length: ∼150 µm.
	Squamata (lizards, snakes)	*Snakes*: Prominent neck cylinder separating nucleus from midpiece; dense body lateral to the centriole. *Lizards*: Single perforatorium from apex of subacrosomal coneElectrolucent region around nucleusFibrous sheath extends into the midpiece. Abundant mitochondria arranged concentrically in large midpiece; irregularly distributed intermitochondrial dense plaques. Sperm length: 80–160 µm.
	Crocodylia (crocodiles, alligators)	Inconspicuous neck. Dense body lateral to centriole. Both linear and concentric mitochondrial cristae, different from lizards and snakes. Sperm length: 80–100 µm.
Birds	Nonpasserines (ratites and some Neognathae)	Filiform sperm, similar to those of reptiles. Perforatorium, proximal and distal centriole. Short midpiece with prominent annulus. Sperm length: 60–230 µm.
	Passerines	Head is twisted spirally, similar to corkscrew; short nucleus. No perforatorium, proximal centriole, and annulus; mitochondria fuse forming single helical structure around axoneme (mitochondrial helix). Dense outer fibrous sheath is prominent and uniform. Sperm length: 40–290 μm.
Mammals	Protheria (monotremes: platypus, echidna)	Sperm similar to typical reptiles and some birds. Long, elongated, filiform, helical head, with 3–5 turns; small acrosome. Tail inserts into caudal end of head via small neck. Short midpiece with mitochondria. Axoneme (9 + 2) with 9 small dense fibers surrounding it. Sperm length: ∼110–120 µm.
	Metatheria (marsupials)	Head shape reflects nuclear shape, generally. *Australian marsupials* have heads with bilateral symmetry. Head resembles wedge, tapering rostrally, with prominent midventral gutter accommodating neck and part of anterior midpiece (except Phascolarctidae). Tail inserts in center or ∼2/3 of ventral surface. Bandicoots and vombatoids have hook-shaped head. *American marsupials* have asymmetric sperm (except for the Chilean monito del monte, *Dromiciops gliroides*, which has structure similar to many Australian marsupials). Small button-shaped acrosome on anterior-dorsal surface of head in some species; acrosome resembles thin layer over dorsal surface in others. Wombats and koalas have bent connecting piece and distal insertion to head. Evidence of equatorial segment and posterior ring in marsupials; no equivalent to eutherian postacrosomal sheath. Flagellum connects via long articulated connecting piece to indentation or implantation fossa centrally located in head (except in shrew opossums, koala, 3 wombat species). Sperm length: ∼80–350 µm.
	Eutheria	Head shape reflects nuclear shape, generally. Head predominantly oval or ellipsoid and symmetric Acrosome generally covers 2/3 of head. Distinct equatorial segment, forming a stable cuff around middle of head, and postacrosomal region. In rodents, some species bear round, symmetrical head, but most have falciform, asymmetric heads. Acrosomes could be very big and extend well beyond the nucleus. Many rodents have apical head appendices (muroid rodents) or basal ones (some caviomorphs).Sperm length: 30–250 µm.

Based on information from the literature (23, 30, 33–56).

##### 2.1.2.1. fishes.

In sharks, skates, rays, and chimeras (Chondrichthyes), spermatozoa are simple (23). The sperm head has a helical shape. In addition to the central axoneme, the flagellum has longitudinal columns. To generate motion, the central axoneme rotates along the flagellum while the longitudinal columns are fixed at positions number 3 and 8 of the doublet. In bony fish (Osteichthyes), the sperm nucleus is elongated and the flagellum contains a flagellar membrane with two lateral extensions resembling two side fins of uncertain function that are thought to improve swimming capacity (36, 50). In teleost fishes, the main group of Osteichthyes and the largest group of vertebrates, spermatozoa do not have an acrosome. This may be linked to the occurrence of internal fertilization and penetration of the oocyte via a micropyle (23).

##### 2.1.2.2. amphibians.

In this group, sperm cells are more filiform and have a perforatorium and a tapering acrosome. The flagellum has a rigid “axial rod” that forms the main axis, displacing the axoneme laterally, and also an undulating plasma membrane traversing the axoneme length, generating propulsion (23). Differences found in spermatozoa of amphibians could relate to differences in the mode of fertilization. Whereas, with a few exceptions, anurans predominantly have external fertilization, the urodeles and caecilians engage in internal fertilization.

##### 2.1.2.3. reptiles.

The reptiles include turtles and tortoises (Chelonia), tuatara (Rhynchocephalia), lizards and snakes (Squamata), and crocodiles and alligators (Crocodylia). They all have internal fertilization and experience decoupling between insemination and fertilization, which leads to long sperm storage times. The sperm cell in reptilians is curved and filiform. There are many differences between reptile species, within and among orders, in relation to the presence of a compartmentalized acrosome complex or the structure of the midpiece (57–60). The midpiece has abundant mitochondria, and the principal piece contains a 9 + 2 axoneme, which is surrounded by electron-dense fibrous sheaths that resemble those in higher-order mammals. Sperm cells vary in length among the four orders (23, 45, 60).

##### 2.1.2.4. birds.

Birds are peculiar in that they control body temperature (as do mammals) and that their body temperature is high. In birds, testes are located inside the abdomen, and therefore spermatogenesis takes place at high temperatures in comparison to the situation in other vertebrates. In general terms, extragonadal storage is less prevalent in comparison to other taxa such as mammals, and sperm cells are released from the testes during ejaculation. After ejaculation, sperm cells find their way to the female sperm storage tubules and remain there for a period of up to 2 wk before fertilization. Sperm morphology shows diversity among birds. Two major bird groups can be recognized on the basis of sperm morphology: nonpasserines and passerines (songbirds). The nonpasserine birds include the ratites and some members of the Neognathae. The sperm cells in these species are filiform and are similar to those of reptiles. On the other hand, passerine birds have a sperm head with a short nucleus, and the head is twisted spirally, bearing resemblance to a corkscrew. Interestingly, a spirally twisted sperm head, as seen in passerine birds, is also seen in other taxa, such as land snails, but, curiously, they differ in the direction in which the head is twisted. Rotation of the sperm head in birds is clockwise, whereas in land snails it is counterclockwise (61). It is not clear how or why these sperm head forms have evolved and why they are so conserved within taxa. Furthermore, the question arises as to whether the chirality in spermatozoa relates or not to the asymmetry in the body of the animals that produce them (61).

##### 2.1.2.5. mammals.

**FIGURE 3.**
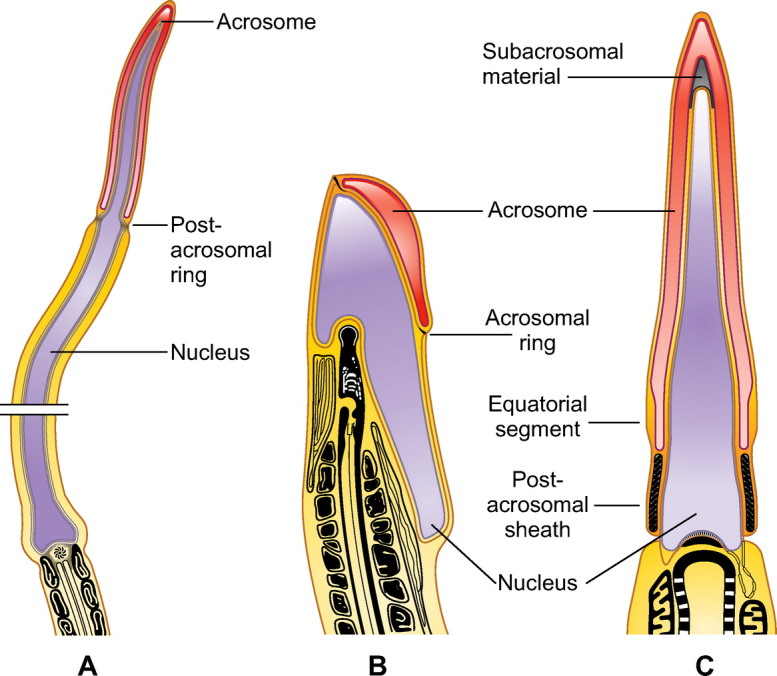
Sperm head structure in mammals. *A*: diagram showing a longitudinal section of equidna (Protheria) sperm head with a very elongated form. Below the acrosome there is a postacrosomal ring. *B*: generalized structure of the marsupial sperm head. *C*: generalized structure of the eutherian sperm head. Modified from the literature (23, 33, 54).

The three infraclasses of mammals (Prototheria, Metatheria, and Eutheria) have sperm structures that are characteristic of each group and specific to each of them (23) (FIGURE 3).

###### 2.1.2.5.1. Monotremes.

Monotremes (platypus and echidnas) derived from therapsid reptiles and are the earliest branching of the mammalian lineage. Platypus and echidna sperm cells are very similar to typical reptiles (and also some bird spermatozoa) (23, 34, 54).

###### 2.1.2.5.2. Marsupials.

In marsupials, the structural organization of the sperm cells is similar to that of eutherian mammals, but spermatozoa have some characteristics that clearly separate them from the latter (54). Different sperm head shapes can be identified in each marsupial family. It is symmetric, resembling a wedge, in most marsupials from Australia. It is asymmetric in most marsupials from the Americas (54). With the exception of shrew opossums (American marsupials), koala, and the three wombat species, the flagellum of all species connects via a long curved or straight articulated connecting piece to an indentation or implantation fossa located centrally in the sperm head. This articulated piece allows the head to rotate on the flagellum, a process that takes place during sperm maturation in the epididymis and that is reversed later, at the time of capacitation in the female tract (23, 54). Marsupial spermatozoa are the longest among mammals, ranging in total sperm length from ∼80 to 350 µm (30).

###### 2.1.2.5.3. Eutherians.

The eutherians are the most abundant group of mammals, with >4,000 species. Rodents are the most diverse and consist of >2,200 species. The general structure of the eutherian sperm cell is conserved, and thus it follows the same basic bauplan, although considerable variations in size, shape, and structural specializations have been reported. It has a sperm head with a nucleus and an acrosomal vesicle, a connecting piece, and a flagellum that is divided into midpiece, principal piece, and end piece, but several structural differences distinguish eutherian spermatozoa from those of other vertebrates and also from monotremes and marsupials.

The predominant sperm head shape in eutherians is oval or ellipsoid and symmetric. In rodents, although some species bear this head shape, the majority show a departure from this pattern, exhibiting shapes that are generally falciform and asymmetric. Many rodent species exhibit sperm head appendices that further enhance the asymmetric nature of the sperm head. The apical extension of the head in muroid rodents is usually known as the hook (or hooks if there are several). Muroid lineages vary in the shape and size of this appendix.

Eutherian spermatozoa vary widely in dimensions. The Chinese hamster sperm is currently regarded as the longest sperm in eutherians, with a total length of ∼250 µm and midpiece and principal piece lengths of ∼100 µm and ∼140 µm, respectively (30). In any case, it is shorter than the longest marsupial sperm and far smaller than the longest animal sperm that is found in the fruit fly *Drosophila bifurca*, in which spermatozoa could be 5.8 cm long, that is ∼20 times the size of the male that produces them (50).

### 2.2. Sperm Functions

Function refers here to how structures are linked to survival and reproduction of cells and, ultimately, of organisms; thus, they relate to mechanisms and processes. In this context, study of function should try to avoid reduction to physics and chemistry and also avoid teleological explanations, although without discarding teleonomic concepts (18, 62, 63). For spermatozoa, functions include survival and the processes related to the ability to reach the site of fertilization and interact with the oocytes. In general terms, spermatozoa are motile cells that swim actively once released to an external medium, or transferred to the female tract, in order to reach the oocytes. In mammals, sperm cells that leave the testis do not yet have the ability to interact with oocytes. They need to experience a series of maturational changes in the excurrent ducts of the male tract that serve to develop the capacity to express active motility. There is an additional series of changes in the female tract that involve modifications at the molecular and cellular levels and that confer the capacity to interact with the oocytes, traverse the cumulus oophorus, experience acrosomal exocytosis, penetrate the zona pellucida, and fuse with the oolemma. There are great losses of spermatozoa in the female tract (or after spawning in external fertilization), and it has been speculated at length as to why are there so many spermatozoa. In mammals, this may relate to the fact that males cannot predict the timing of ovulation and thus it is to their advantage to produce a large, and diverse, number of sperm cells that can respond to signals or be ready to fertilize at different times.

#### 2.2.1. Sperm transport and preparation for fertilization.

Upon release from the male, either at spawning or after transfer to the female tract, spermatozoa begin to move in a process generally known as “activation.” In mammals, spermatozoa are stored in a quiescent (repressed) state in the cauda epididymis after their long transit in the epididymis. Upon contact with fluids that are secreted by the male’s accessory glands, the sperm cells initiate forward progression. There are differences between species because some males ejaculate in the vagina, others in the uterine cervix, and yet others in the uterus (64). This means that there are differences in the fluids they come in contact with and also the barriers they need to negotiate. Furthermore, the timing between mating and ovulation varies widely, and in some species such as bats sperm need to survive in the female tract for a very long time (65).

In the uterus, sperm are moved passively by contractions of this organ, but they need to swim actively through the utero-tubal junction and reach the lower oviductal isthmus, that is, the lower portion of the oviduct (66). It has been experimentally shown that, at this stage, sperm usually associate with the epithelial cells of the oviduct and become quiescent (64). The binding is mediated by species‐specific carbohydrate moieties present on the cilia localized at the apical portion of epithelial cells and the surface of the sperm head (64, 65). Only the sperm that establish this association remain viable (67, 68). The shape of the sperm cell seems to be important in the type of association that is established with the oviductal wall, and, moreover, the sperm has to bear an intact acrosome (i.e., has not undergone the exocytotic process); otherwise it will not be able to attach (69).

This period of residence in the lower isthmus varies among species and is believed to be associated to the time between the onset of estrus (and mating) and ovulation. During this time, spermatozoa experience a series of additional changes that would render them capable of detaching from the oviductal wall by expressing a different pattern of motion and start swimming toward the site of fertilization in the oviductal ampulla. This new pattern of motility is known as hyperactivation because sperm now has a much more active beating pattern when examined under in vitro conditions. Because the oviductal lumen contains fluids that enhance the viscosity of the medium, the role of hyperactivation, which endows spermatozoa with a stronger propulsive capacity of the flagellum, seems to be to allow cells to move forward under these conditions, whereas those that do not develop hyperactivation cannot advance in this viscous fluid (64, 69–71). This hyperactivated motility is also important later on when sperm penetrate the oocyte vestments (72, 73). The development of this type of motion has been the center of much attention, with many studies dealing with its underlying molecular changes (27, 74, 75). Hyperactivation is triggered by an influx of Ca^2+^ through the cation channels CatSper (76). The process may also involve other ion channels and increase in bicarbonate, cAMP, and phosphorylation of specific proteins (74). It is important to note that most of the molecular signaling studies were reported for mouse and human spermatozoa. There is little information for other species, and this is an area of research that deserves further attention. Remarkably, it is also still poorly understood how hyperactivated motility relates to sperm shape in different species.

During their residence in the female tract, spermatozoa develop the ability to interact with the oocyte (77–79), a process known as “capacitation,” although it most likely occurs when the sperm are attached to the oviductal isthmus. There is still controversy regarding the use of the term for the entire period of residence in the female tract (19, 21, 80, 81). Several authors reserve the term just for the changes that prepare the sperm for hyperactivation and acrosomal exocytosis, excluding these latter processes from capacitation (82–87). Capacitation is thought to occur only in mammals, and many efforts to understand the underlying molecular mechanisms have been carried out, with several recent reviews representing a good source of information on this process (1, 81, 88–90). It is well accepted that during capacitation there are biophysical modifications in the plasma membrane as a result of increased concentration of $\mathrm{HC}O_{3}^{-}$ and removal of cholesterol by albumin. The initial influx of $\mathrm{HC}O_{3}^{-}$ activates adenylate cyclase (ADCY10), leading to increase in cAMP, PKA activation, and subsequent protein tyrosine phosphorylation. Several regulatory and cross talk mechanisms interact during this process, and they are not yet fully elucidated. It has been shown that PKA is essential for CFTR activity and for sustained increase in $\mathrm{HC}O_{3}^{-}$. Additionally, the membrane depolarizes and ion transporters are activated to regulate influx and efflux of ions. Ca^2+^, K^+^, Na^+^, and H^+^ are the main cations transported, and Cl^−^ and $\mathrm{HC}O_{3}^{-}$ are the main anions (90). It is remarkable that since the discovery of capacitation by Austin and Chang 70 years ago there are still many unknowns around its essential molecular signaling. Although some of the ion channels and transporters have been identified in the sperm, there are still more unknown steps as well as participating players. Moreover, our knowledge about the sequential activation of events is still rather rudimentary. Because capacitation is an essential event for fertilization, further efforts, resources, and cutting-edge technology should be invested to advance in the understanding of this molecular mechanism. Furthermore, it may also be rewarding to ask how spermatozoa are programmed in the testis, or modified in the epididymis, to be able to undergo the process of capacitation.

Additional mechanisms have been described to occur before fertilization. For instance, in species with internal fertilization, several studies have reported the participation of sperm-guiding mechanisms that move the spermatozoa toward (rheotaxis, thermotaxis, and chemotaxis) or away from (chemorepulsion) the egg surface (66). The first are important for the gametes to meet, and the latter may function as a way to avoid polyspermy. Unfortunately, the molecular signaling for these mechanisms has not yet been extensively studied, and there are only a few reports dealing with their molecular signaling in mammals (91). Regarding the chemotactic signaling, it has been shown that human spermatozoa respond chemotactically to a picomolar gradient of progesterone after binding of progesterone to its cell surface receptor. This initiates the activation of a transmembrane adenylate cyclase (tmAC)/cAMP/PKA pathway followed by protein tyrosine phosphorylation and Ca^2+^ mobilization [through inositol (1,4,5)-trisphosphate receptor (IP_3_R) and store-operated Ca^2+^ (SOC) channels]. The soluble guanylate cyclase (sGC)/cGMP/PKG cascade is activated later, and a possible further Ca^2+^ influx through another plasma membrane calcium channel occurs (92). The chemotactic signaling was also investigated using bourgeonal, an aromatic compound, as a chemoattractant (93). These authors also reported the participation of the tmAC/cAMP/PKA pathway and subsequent tyrosine phosphorylation of proteins. Additionally, the sGC/cGMP/PKG pathway has been proposed to underlie human sperm chemotaxis in response to nitric oxide donors (94). Despite many recent advances, there are several questions that wait to be answered. For instance, among others, What is the identity of the progesterone receptor? What other ions in addition to Ca^2+^ participate in this signaling? What other channels and/or transporters participate in the signaling? What are the proteins phosphorylated, and where do they localize in the sperm cell? What other molecules are involved? What is the sequential activation of the events? And most importantly, is this signaling conserved among species?

No major structural changes appear to take place in the sperm during capacitation, On the other hand, the subsequent step of exocytosis of the acrosome involves a major structural reorganization, because this organelle occupies a considerable part of the head (2). Fusion at multiple points between plasma membrane and underlying outer membrane of the acrosome results in massive release of enzymes through the pores and, more importantly, the final detachment of the acrosomal cap. This results in an overall change in the structure of the cell. Importantly, the release of the acrosomal cap in many species, with significant modification in sperm head shape, could have implications for sperm hydrodynamic efficiency in the final steps of penetration of the cumulus oophorus and the zona pellucida. The acrosome reaction signaling has been intensively studied over the last two decades. Excellent articles reviewing the vast bibliography in this area have recently been published (89, 95, 96). The working model for this signaling involves Ca^2+^ as the key cation transported, activation of adenylate cyclase, EPAC, RAB family proteins (Rab3, Rab27), PLC, and fusion of membranes through the soluble *N*-ethylmaleimide-sensitive factor (NSF)-attachment protein receptor (SNARE) complex (97). There are also recent summaries of the sequential activation of the signaling cascade (95).

#### 2.2.2. Bioenergetics.

Spermatozoa require energy to maintain homeostasis and cell integrity, signaling, and, above all, to sustain motility, a function that can consume >75% of all energy produced (98, 99). In species with external fertilization, spermatozoa do not draw energy substrates from the medium and rely on endogenous reserves (100). In many species, sperm survive for a short period of time and thus must encounter the oocytes quickly. Interestingly, sperm from some animal species that live in habitats with very low oxygen levels lack mitochondria (101). In species with internal fertilization, spermatozoa can draw from energy substrates present in seminal plasma or in various regions of the female tract (102, 103).

Species differ in metabolism and can produce ATP via glycolysis, oxidative phosphorylation (OXPHOS), or both pathways (104–106). Among mammals, bovids appear to use both pathways, cats generate the majority of ATP via OXPHOS, and human spermatozoa employ glycolysis for energy production (104–106). Stallion spermatozoa require ATP generated by mitochondria to maintain membrane integrity, whereas they need both glycolysis and OXPHOS for motility (107). In rodents, there are differences between taxa. In the guinea pig (which belongs to the caviomorph group), spermatozoa produce ATP via both glycolysis and OXPHOS. In the mouse (which is a myomorph rodent), studies in laboratory strains have concluded that ATP production takes place mainly by glycolysis, but more recent studies on wild-derived mice revealed that the situation is more complex. Thus, among closely related mouse species, production of ATP may vary, with energy being generated mainly via glycolysis in some but in others OXPHOS seems to make significant contributions (108). The levels of ATP are crucial for sperm swimming performance. This is underscored by the finding that, across species, there is a significant positive relationship between the amount of ATP in sperm and sperm velocity (109, 110).

It is also relevant to consider how sperm consume ATP. Studies in rodents showed that there are differences in how sperm from different species use ATP, independent of its production. The pattern of ATP utilization was found to have an effect on sperm performance, including swimming efficiency (111). Sperm energy requirements may also vary during the life of the sperm cell, but little information exists on this matter. Furthermore, sperm cells may resort to different metabolic pathways in different physiological states (102, 112). Thus, it could be speculated that spermatozoa that have undergone capacitation and show hyperactivated motility could have different energy demands. It is tempting to propose that with a more vigorous movement, such as that observed during hyperactivation, sperm may require more ATP. Whether spermatozoa produce more ATP or use it more efficiently, and what the pathways to generate it are, remain a matter for future research.

### 2.3. Heterogeneity in Form and Function

Despite a general pattern of shape and size that can be recognized for spermatozoa of each species, there is heterogeneity in both traits even for a given male. Thus, there is variation both between and within a species and, in addition, within an individual male, which is reflected in the heterogeneity of sperm released or ejaculated. This heterogeneity in shape and size will impact on sperm function. Variation at these different levels may result from a number of factors and may have various adaptive advantages. There may be differences during the process of sperm production, in posttesticular maturation and storage, and also in how (and when) sperm are released or transferred to the female tract. For example, spermatogonial stem cells form syncytia, and in rodents 1,000 or more spermatogonia can be interconnected and undergo meiotic divisions synchronously, resulting in ∼4,000 connected spermatids undergoing differentiation; the magnitude of mitotic amplification varies between species, being 128- or 16-long syncytia in monkeys and humans (13). There is heterogeneity in the transcriptional states (gene expression) of spermatogonia that relates, in part, to the length of the syncytia (113). Studies of behavior of individual cells have revealed that individual stem cells have variable fates with regard to the number of self-renewing and differentiating cells in a cohort derived from a single spermatogonial stem cell (114). Fate variance among clones would occur over time (clonal drifts) and may result in the eventual domination by a single clone, thus resulting in “population asymmetry” (113, 114). It follows that heterogeneity of an ejaculate would have to take into account that it could be the product of competing germ cell clones.

Another example relates to epigenetic heterogeneity. Epigenetic modifications influence the function of male germ cells, and alterations in levels of DNA methylation are known to be associated to sperm abnormalities, decreases in fertilization rates, and deficient embryo development (115). In human sperm, evidence for heterogeneity in DNA methylation of one maternally methylated gene (*KCNQ1OT1*) has been found in samples from men with abnormal sperm parameters (116). Sperm from normozoospermic samples had a homogeneous pattern of DNA methylation, but in samples with low sperm motility (oligoasthenozoospermic men) there are discrete groups of spermatozoa with either normal or abnormal patterns of methylation (116). On the basis of these findings, and additional information, possible scenarios in which spermatogonial stem cell variability can lead to sperm epigenetic heterogeneity have been presented (115). The relative importance of internal factors or those from the environment, and their impact on fertility, for this heterogeneity deserves additional attention.

Heterogeneity may also arise as result of postmeiotic gene expression, and this may lead to sperm within a single ejaculate being subject to selection. The idea that postmeiotic expression of genes may result in unequal sperm has generated controversy (117–121) because gene transcripts and proteins may be fully shared among cells during sperm formation because of cytoplasmic bridges among haploid spermatids (122–126). There is, however, evidence for unequal sharing of some gene products in the mouse (127–129) and a link between sperm genotype and sperm phenotype in zebrafish and human (130, 131). The extent of specific haploid transcription is unclear (129, 132), and it may even take place in a mitochondrial-type ribosome (133). Because haploid gene expression may bear on the sperm phenotype (e.g., form or function), this may be the basis of haploid selection in a single ejaculate, as revealed by differences in zebrafish short- versus long-lived sperm that were related to differential offspring fitness (126, 130) This is to be distinguished from selection by competition between ejaculates from different males (see below).

A special case of sperm diversity based on haploid gene expression relates to possible differences in phenotype due to expression of X or Y chromosome-borne genes. In mammals, there is heterogeneity with regard to sperm bearing either an X or a Y chromosome, and it is thought that about half of sperm in an ejaculate bear each type of chromosome. As shown in the mouse, sex chromosomes have a very high level of copy number amplification of postmeiotically expressed genes (134, 135). This situation is different from that in birds, in which all the sperm carry a Z chromosome, since males are the homogametic sex in these taxa. Biases in sex ratios exist in mammalian offspring, and it is thought that either females may chose one type of sperm over another or males are capable of producing, or releasing, more sperm carrying one or the other type of chromosome (136, 137). Evidence is still controversial as to the possible underlying mechanisms mediating biases in sex ratios. One example has been identified in mouse sperm in which Yq-deleted mouse males (MF1-XYRIIIqdel) produce equal numbers of X- and Y-bearing sperm, but such sperm show variant morphologies and are functionally different from each other, exhibiting motility differences that would explain offspring sex ratio due to differential transport in the female tract (138). Thus, postmeiotic differential gene expression between X- and Y-bearing sperm may influence sperm head size or shape, swimming performance, surface proteins, other aspects of sperm function, or a combination of these traits (e.g., relations between head shape and hydrodynamic efficiency) (138–141), making them more susceptible to external factors or of being differentially selected in the female tract.

Heterogeneity in spermatozoa has been categorized and quantified both within and between males in order to understand sperm subpopulation structure and possible relationships with fertilizing ability (142, 143). Differences in subpopulation structure between males, based on sperm size and shape, have been identified for several species of domestic animals (144–147). Such distinctions are relevant for male fertility because a clear relationship between the proportion of sperm forms in a given subpopulation and fertility of the males has been found (142). Similarly, subpopulation structure based on sperm motility (kinematic parameters) has been characterized in domestic species (148–151) and relationships between proportion of sperm in a population and male fertility uncovered (143). Very few studies exist in which shape and motility parameters have been combined to characterize sperm subpopulations (152), and to the best of our knowledge there are no reports yet on links between a combined analysis of shape and motility subpopulations and sperm fertility. As a cautionary note, it is perhaps important to bear in mind that diversity in sperm morphology and kinetic traits (or lack of them) may be related to methodologies employed and there is a risk of generating artifacts during analyses (reviewed in Refs. 19, 153–155). In any case, the question that arises is why males produce not only vast numbers of spermatozoa but also these heterogeneous populations if only a few sperm cells would be successful at fertilization.

## 3. EVOLUTION OF MAMMALIAN SPERMATOZOA

Diversity in spermatozoa has generated considerable interest. Many efforts have been directed toward understanding patterns of evolution of sperm cell morphology and function. Significant interest has also been placed in trying to understand the selective pressures underlying such diversity. This section therefore deals first with the patterns of evolution of spermatozoa and second with evolutionary forces that may explain sperm diversity.

### 3.1. Evolutionary Patterns in Spermatozoa

From a biological point of view, males are defined as the sex that produces the smaller gametes (i.e., spermatozoa), as opposed to females, which are those that produce the larger gametes (i.e., oocytes) (156, 157). It is thought that, ancestrally, only small monomorphic gametes were produced (158). Therefore, an important question is what pressures have caused gamete dimorphism (anisogamy) to evolve in such a crucial transition (159). Divergence into two sexes seems to be an (almost) inevitable consequence of sexual reproduction, and it seems to be driven by gamete competition (160). The subject of early divergence of two gametes of different sizes and morphologies is beyond the scope of this overview. The reader is referred to recent reviews that cover several aspects, including modeling, of the evolution of anisogamy (160–163) and how it integrates in the so-called sexual cascade (164).

Early studies focusing on evolution of sperm attempted to draw general patterns for the entire animal kingdom or concentrated on species with external fertilization (165–169). Species with external fertilization, which release gametes into the water, are believed to have sperm cells that are generally simple in their morphology, with a round head and a flagellum of ∼50 µm in length with a 9 + 2 axoneme (Refs. 170, 171, but see Ref. 172). On the other hand, in species with internal fertilization, spermatozoa are more complex and have longer flagella (170). Additional changes related to internal fertilization may be a reorganization of mitochondria and, in mammals, the appearance of accessory fibers in the flagellum that probably help when swimming in the more viscous fluids of the female tract (173–175).

Studies of evolutionary patterns of mammalian sperm have focused on two main aspects, one addressing changes in sperm size in relation to body size (that is, possible allometry), together with scaling of the different sperm components, and the other trying to understand patterns of sperm head change in different animal lineages. In addition, the significance of changes in sperm length or variation of sperm head shape in relation to sperm swimming ability has also received consideration. These issues are considered next.

In mammals, sperm size varies widely. Although relatively small differences exist in sperm head length, differences in flagellum length are considerable between species. Overall, marsupial sperm are much longer (range: 80–350 µm) than eutherian sperm (30–250 µm) (30, 176). Among eutherians, rodents are the species with the widest range (35–250 µm) (30, 176). Proportions of the different sperm components also vary; for example, comparison between marsupials and rodents shows differences in the sperm head (6% vs. 7.5% of total length, respectively), midpiece (11% vs. 23%), and principal piece (83% vs. 70%) (30). Although early studies in mammals proposed that sperm size (and size of its components) was inversely related to body size (30), subsequent studies revealed that the relationship is built on extreme values, with large mammals having small sperm and small ones having, in general, longer spermatozoa, and that there is no significant relationship between body size and sperm length (177). In fact, sperm size is rather uniform across many mammalian orders, and it is only in some groups (marsupials, rodents) in which a wide variation in sizes is observed (30, 178). With regard to different sperm components, it seems that there is a positive association between lengths of various sperm parts, with a proportional increase of different components (177). Sperm midpiece and principal piece lengths are positively associated with both head length and area, and principal piece length is positively associated with midpiece length (177). Interestingly, sperm head dimensions are not related to either genome mass or chromosome number (177).

Diversity in sperm head shape has been examined in rodents in order to understand the direction of evolution, using a combination of phylogenetic trees based on genetic, chromosome, morphological, and biogeographical data together with sperm morphological information. These analyses revealed that in several rodent lineages, including caviomorphs (the group that includes guinea pigs, among others) and myomorphs (the group with mice, voles, hamsters, South and North American cricetids, and African nesomyids), there are spermatozoa with a simple, round-oval head shape and short flagella. Each lineage also shows species with a peculiar sperm head that becomes more complex (and in some cases includes more apical appendices) as sperm becomes longer. This pattern led to the suggestion (176) that, in rodents, ancestors for each lineage had a simple sperm head, with a short flagellum, and that an increase in complexity and length has occurred in different lineages, with each lineage developing different head morphs. Thus, increases in sperm complexity involved the repeated appearance of an asymmetric, falciform head, with the development of hooks or the displacement of the site of flagellum insertion in the base of the tail. Such a view envisioned that the diversity in sperm forms was related to adaptive advantages for spermatozoa (176).

An alternative interpretation of rodent sperm evolutionary patterns considered that the ancestral sperm morphology for myomorph rodents was an asymmetric, falciform one, with a hook in the apical end of the sperm head, from which more complex heads had evolved. In this view, the round-oval, paddlelike sperm shapes seen in myomorph rodents were interpreted as being derived morphs, rather than the ancestral ones (179, 180).

**FIGURE 4.**
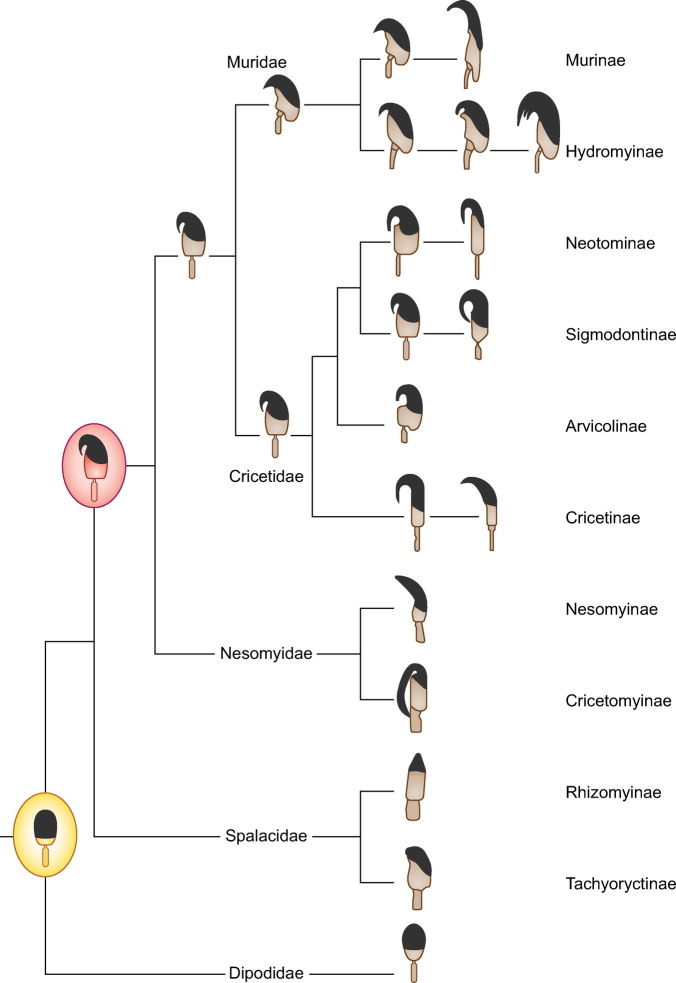
Evolution of sperm head shape in muroid rodents. A model for the evolution of sperm head shape in muroid rodents. Spermatozoa in different lineages show specific patterns, with an increase in complexity, which is accompanied by an increase in length (not shown). A simple, symmetric, round or oval sperm head was probably present at the base of the muroid evolution at the time when divergence of these lineages occurred 25–30 million yr ago. An elongated sperm head with an apical hook appears to have evolved in a common ancestor of the Nesomyidae, Cricetidae, and Muridae, with this sperm form being common throughout the nesomyid, cricetid, and murid rodents. Based on results in Roldan et al. (176) and Breed et al. (181).

The controversy was resolved when the range of myomorph rodents examined was expanded (181). Spalacid species, a family of the superfamily Muroidea from eastern Asia, the Horn of Africa, the Middle East, and south-east Europe, were found to have simple, symmetric, ellipsoid sperm heads without a hook. They, together with the Dipodidae, seem to represent the oldest split in the muroid superfamily, and thus the common ancestor with the rest of Muroidea probably had this type of simple, symmetric sperm head (181). The reorganization of the sperm head, with the development of asymmetry and an apical hook, probably arose in a common ancestor of mice, cricetids, and nesomyines (FIGURE 4). Furthermore, there seems to be coevolution of head shape and sperm size, because the more complex or elongated rodent sperm heads are seen in species with the longest spermatozoa, and this is true for different rodent lineages (176). In any case, there remains the issue of the repeated reversal of the complex sperm head trait to a simpler head pattern with regard to both mechanisms and evolutionary pressure (or lack thereof).

The evolution of longer sperm could be adaptive, because longer sperm may swim faster (182). Empirical evidence for this idea was first presented in a comparative, interspecific study of mammals (182, 183) and then expanded to other taxa (reviewed in Ref. 184). In rodents, sperm swimming velocity is associated with the size of all sperm components (185, 186). In addition, sperm head shape also impacts on swimming velocity. In a study across mammals, sperm with more elongated heads were found to have higher straightline velocity, i.e., they swim faster (183), whereas in rodents changes in the sperm head, including the appearance of the hook, are associated with increased overall sperm velocity (186, 187).

Changes in sperm morphology (following the appearance of a hook in the sperm head) may be linked to the development of associations between spermatozoa (that is, the formation of the so-called “sperm trains”) and display of faster swimming velocity. The phenomenon of association of sperm cells and swimming in trains, with accompanying faster sperm, was described in rodent species *Apodemus sylvaticus* and the genus *Peromyscus*, and detailed characterization of such swimming behavior has been presented (188–190). The associations observed in rodent sperm resemble somehow those observed in sperm from American, but not Australian, marsupials (191, 192), although in these species pairing of sperm occurs in the epididymis (193), in contrast to the associations in rodent spermatozoa, which seem to occur after ejaculation. Marsupial sperm also swim in pairs when migrating in the reproductive tract. It has been postulated that this behavior may serve to protect the acrosomes (because sperm heads are joined together over their flat acrosomal surface), but most likely it results in paired spermatozoa swimming faster in the viscous environment of the female tract (194). This phenomenon should also be distinguished from sperm associations that occur in the epididymis, during sperm maturation, and that result in ejaculation of sperm groups (e.g., guinea pig rouleaux) (195).

Because forward-moving trains result mainly when sperm cells associate by the heads, it has been speculated that head hooks could have an important role in this phenomenon and, furthermore, that they have evolved in connection to this swimming pattern (Ref. 196, see Ref. 197 for additional references). However, an analysis of several rodent species without or with a head hook has not shown a consistent pattern of train formation and presence of an apical appendix (197). Therefore, it seems that train formation is an exceptional strategy for faster sperm swimming that appears to have evolved in parallel in a limited set of species. A thorough study is needed to characterize sperm structures involved in these sperm associations and to understand the swimming patterns of these associations as well as the kinetics of train formation and sperm release from such trains in relation to different stages of sperm transport in the female tract.

At the intraspecific level, analyses of red deer spermatozoa showed that cells with elongated heads swam faster, and a similar association was found for the proportion of the principal piece in relation to the length of the flagellum; in contrast, a negative relation was observed between midpiece length and swimming velocity (198). In mice (199), an intraspecific comparison revealed a relationship between sperm form and sperm function, and in this case midpiece length was found to predict swimming velocity in lines bred under different selective pressures. Explanations for this apparent discrepancy invoked differences in sperm head shape between these species, but, since swimming is also influenced by bioenergetics, it is possible that sperm from deer and mouse are obtaining ATP via different pathways, which may influence sperm performance together with cell morphology (109). In addition, differences in sperm head shapes between these species may have different impacts on drag (200).

In birds, it was predicted that increased sperm velocity may be associated with an enlarged midpiece (because of its energetic role) or overall flagellum length (because of the propulsive force it generates) or the proportion between sperm components. A comparative analysis in passerine birds revealed that sperm velocity significantly associated with sperm dimensions in the predicted direction (201). Similar results were obtained in intraspecific studies in zebra finches, in which sperm velocity was positively correlated with sperm length; because both were found to be heritable, selection for faster sperm will simultaneously lead to the evolution of longer sperm, and vice versa (202). The effect of length on sperm velocity is also suggested by the observation that spermatozoa with longer flagellum lengths preferentially reach the site of fertilization in zebra finches (203). In this species, a trade-off between length and thickness of the sperm midpiece was reported, with longer midpieces being proportionally thinner with, interestingly, similar mitochondrial material, which can have implications for energy production (204). In intraspecific comparisons in the house sparrow, sperm velocity was found to be correlated with head-to-flagellum length ratio: sperm with small heads relative to their flagellum showed higher swimming velocity, which is consistent with the idea that less drag (because of a smaller head) and more propulsion (because of a longer flagellum) make for more efficient swimming (205).

The shape of the sperm head was revealed to be important for passerine birds. Spermatozoa with heads that have a more pronounced helical form (with long acrosome, short nucleus, wide helical membrane, and a more pronounced waveform along the sperm head) exhibited faster swimming (206).

### 3.2. Evolutionary Forces Underlying Diversity in Sperm Bauplan and Function

Once the patterns of evolution of sperm cells are recognized, including direction of changes and some reversals of complex traits, it is possible to ask what selective forces may underlie them. Importantly, inquiring about selective pressures is one of several types of questions that can be asked to understand diversity in sperm cells. As recognized by Mayr (62) and expanded by Tinbergen (207), causality in biological systems could distinguish ultimate or proximate questions. Whereas evolutionary explanations address ultimate causation, that is, distant in time, proximate causation addresses immediate factors, close in time, such as physiological mechanisms. This distinction is important and allows us to ask “why” and “how” questions separately, with different toolkits, in an effort to avoid confusing levels of analyses. Among the evolutionary explanations, attention is given to either adaptive value or phylogeny (history) of traits. Included in the proximate explanations are either the mechanisms or the development (changes during one or more stages in life of individuals) of traits (208–211). Integration or complementation of levels of explanation is certainly desirable because separation of such levels may limit advances in our understanding of biological systems (212, 213). Thus, complementation of questions regarding evolution and development has given rise to the field of evolutionary developmental biology, which allows us, for example, to focus on issues of developmental constraints or reciprocal causation (208, 209). These aspects, and an initial attempt to relate them to sperm biology, have been discussed elsewhere (18).

Whereas sect. 4 considers mechanisms of sperm formation to identify processes that may help us understand the origin of biological novelty and diversity in morphology and function, the rest of this section deals with evolutionary questions. Three main selective pressures are considered to understand evolution of morphology and function of spermatozoa and, in turn, processes of sperm formation in the testis. One relates to mode of fertilization (or fertilization environment), another to postcopulatory sexual selection, and yet another to common descent or phylogeny (214).

#### 3.2.1. Mode of fertilization.

This factor has probably exerted an important influence on the evolution of gametes. Vertebrate species differ in their mode of fertilization and, hence, in the way their gametes meet and interact. Internal fertilization takes place in all elasmobranchs (sharks, rays, skates, sawfish), some oisteichthyans (bony fishes), all gymnophione amphibians (caecilians), one species of anuran amphibian, most urodele amphibians (salamanders), and all amniotes (reptiles, birds, mammals) (32). In externally fertilizing species, the structure of the sperm cell is thought to be rather simple (170). Because water is a uniform environment, spermatozoa have to swim to reach the oocyte, and such simple structure is adaptive under these conditions. Internal fertilization is believed to have led to a series of modifications generating a longer and more complex sperm cell (48, 171). A recent comparative analysis of >4,000 vertebrate species has indeed found robust evidence that longer spermatozoa have evolved in species with internal fertilization (215, 216).

In internal fertilizers, sperm cells have to swim from the site of deposition, usually a cloaca, urogenital sinus, or vagina, although in some species they are placed directly into the uterus or part of the oviduct (69). The distance to swim may vary from a few millimeters in small species to a meter or more in some large species such as elephants or whales. Although contractions of the female tract are important in some species, forward progressive sperm motility is a key requirement. Sperm traits have been found to relate to size of the female reproductive organs or time spent in the female tract (217, 218).

Much of the analysis on mode of fertilization has focused on the impact it has on the interaction between sperm and oocyte (73). In mammals, the extracellular oocyte coats (or vestments) have increased in size. The cellular coat (the cumulus oophorus), which is the first vestment that the sperm has to penetrate, is a multilayer of cells that surrounds the oocyte and has an abundant matrix that would slow sperm in its transit. The acellular coat (the zona pellucida) has become thicker in eutherian mammals, and thus the interaction of the sperm cells with this structure has resulted in several changes in the spermatozoon. Sperm need to generate more force to penetrate the zona, and to this end the sperm head has flattened and, in addition, an apical structure with a more resistant structure has appeared: a perforatorium with a scythelike shape is present along the rostrum of the head, adjacent to the nucleus. Both the flattened head and the perforatorium may serve to cut a path through the zona pellucida when the spermatozoon tries to gain access to the oocyte (73). Because exocytosis of the sperm acrosome exposes the inner membrane of the granule, the head has also experienced modifications in the region of the sperm that will fuse with the oocyte’s plasma membrane. In addition, because penetration now requires stronger propulsive forces, a change in the pattern of movement that takes place during sperm capacitation (i.e., hyperactivation) contributes to more vigorous movements that help in the path to zona penetration and also contributes to the change in form and reorganization of subcellular structures.

In species with internal fertilization there may be additional influences due to differences in their reproductive mode, that is, whether species lay eggs (oviparity) or bear young eventually leading to live births (viviparity). In a comparative study in snakes, oviparous species were found to have longer spermatozoa than viviparous species (56), a result that was confirmed in a larger sample across vertebrate species (215). The reasons for this effect are not entirely clear. It could be that, since cost of reproduction may differ between oviparous and viviparous species, postcopulatory selection may also vary. It is also possible that differences are related to considerable variation in mechanisms of sperm storage and transport between these taxa and, moreover, in the overall morphology of the female reproductive tracts and temporal differences in ovulation and fertilization. Such differences can impact on sperm physiology and, in turn, on sperm bauplan. In oviparous species, such as birds, eggs are released and fertilized in sequence, whereas in viviparous species eggs are released and fertilized simultaneously. This would most likely generate completely different scenarios for males and their sperm (219).

#### 3.2.2. Postcopulatory sexual selection.

A set of selective pressures, collectively known as postcopulatory sexual selection, has been invoked to understand variations in sperm shape, size, and performance. These pressures have been named in correspondence to the sexual selection forces that take place before mating (220) and that are thought to explain certain adaptations that are not linked to survival but to reproductive success (164, 221). Sexual selection involves modes of selection in which members of one sex choose members of the other sex for matings (intersexual selection) or members of one sex compete among themselves to gain access to the opposite sex (intrasexual selection) (21, 222). It has been found that these pressures lead to the development of certain sexual characters that represent advantages in competition, such as body size, the size or shape of antlers or horns, or coloration. When females choose, they can do it on the basis of their preference for a given male trait. When these selective forces operate after mating, they are known as postcopulatory sexual selection (21). Selection by females manifests as a process of cryptic female choice in which they may select one type of sperm cell over another. The competition between males to gain access to females takes the form of competition between spermatozoa from rival males (sperm competition), with the aim of achieving fertilization of oocytes. Altogether, these phenomena may be interpreted as either females choosing particular males or sperm with particular attributes or relatively passive females accepting the winner of fights among males or their spermatozoa. However, on a more general level, and taking into account that in some species females are more brightly colored or aggressive than males, it matters, for a more general formulation of the principle of sexual selection, which is the sex with the higher or lower potential reproductive rate instead of male or female (222).

Cryptic female choice arises from female-driven mechanisms during or following mating that bias sperm use and have an impact on the share of male paternity (73, 223). Several aspects of female biology (behavior, morphology, physiology) exhibit potential for cryptic female choice during mating, sperm transit or storage, through to sperm-oocyte interaction, biasing fertilization toward the sperm of specific male or males (224). Nevertheless, until now it has not been clearly demonstrated, probably because it requires distinction of components of male and female variance that contribute to sperm retention and paternity; thus, much remains as theoretical speculation (224). With regard to its adaptive value, under certain conditions, cryptic female choice could have implications for female fitness or sexual conflict (225, 226).

To gain evidence for cryptic female choice it may be necessary, for instance, to identify a female trait that influences sperm retention or utilization after mating and that the female response is nonrandom in a way that results in sperm of certain males being differentially favored based on genotype or phenotype (224). There are examples of sperm ejection by contractions of the female tract, which thus limit transit from lower sections of the female tract (227). Females may also influence sperm storage based on the degree of relatedness to males (228) or affect sperm swimming performance via reproductive fluids, including processes of chemoattraction (225, 229). Finally, there is also the possibility of choice at the time of oocyte-sperm interaction either in relation to inbreeding avoidance (230) or promoting a given major histocompatibility complex haplotype (231). In mouse species, adaptation to the risk of polyspermy may influence oocyte defensiveness, which may serve to filter and select spermatozoa on the basis of compatibility or quality (232).

Sperm competition happens when females are promiscuous and mate with two or more males during their period of sexual receptivity (16, 233). In the case of external fertilization, sperm competition takes place when spermatozoa from several males are released more or less simultaneously and they attempt to gain fertilization of oocytes that are released concomitantly (234). Although many studies have focused on the evolutionary framework of sperm competition (5–7, 48, 235), there is still little knowledge regarding the underlying mechanisms that influence sperm competitive ability (221, 236).

In recent decades there has been much interest and effort in trying to elucidate the impact of sperm competition on evolution of spermatozoa, both with regard to their numbers, shape, and size and also in relation to function. The conclusions obtained from much work suggest that an increase in levels of sperm competition results in an increase in relative number of spermatozoa, that is, the number of spermatozoa corrected by body mass (because larger males will produce more sperm to compensate for larger female tracts); sperm numbers evolve together with increases in relative testis mass (222).

There are also changes in the shape of the sperm head in response to sperm competition, because this selective force seems to promote elongation of the sperm head in mammals (183). The adaptive significance of modifications in shape relates to the realization that sperm with more elongated heads may swim more efficiently (183). Other modifications in the sperm head are difficult to associate with sperm swimming, among other reasons because of the difficulties of quantifying shape (19, 21, 237). The evolution of appendices such as hooks modifies head shape and renders them more asymmetric, so inference of shape from head dimensions, as done in studies with simple, symmetric sperm heads, is not reliable. Analyses of the curvature of hook insertion in the main part of the head, or the length of the hook, have revealed that both are positively associated to levels of sperm competition in rodents (187, 196). Changes in hook angle or length probably influence the hydrodynamic efficiency of sperm head shape.

The association between sperm competition and sperm size (or proportions between the lengths of sperm components) has been observed in many taxa in both inter- and intraspecific comparative analyses (21, 182, 184). There are some exceptions to this trend, which can perhaps be explained by other selective forces (fertilization mode or other reproductive patterns). In any case, a vast majority of comparative analyses, in many animal taxa, have revealed a positive association between sperm competition and length of spermatozoa (238). The influence of sperm competition on sperm numbers (see above) has led to the proposal that in order to produce more sperm males would reduce sperm size (239). However, it has been shown that this may not be the case, at least in mammals, because higher levels of sperm competition also result in longer spermatozoa and both numbers and dimensions may coevolve positively (240). Sperm competition influence on sperm size reflects positively on sperm velocity (184, 201).

There is also a direct association between sperm competition and sperm swimming velocity (186, 201). It is possible that sperm competition influences sperm velocity via processes other than (in addition to) the effect of sperm length. For instance, changes in sperm energy production and consumption may also impact on sperm performance. Although relatively little is known about how sperm from many species produce their ATP, some studies have used midpiece length or volume (where mitochondria reside) to examine whether sperm competition influences sperm metabolism (241–243). Comparative analyses of actual ATP levels have revealed differences in the amount of sperm ATP in rodents from several lineages (mice, voles, hamsters) and a positive relationship with sperm competition levels (110). Considerable differences were also found in a comparison across mammalian species, and, again, a positive association with sperm competition was uncovered (109).

Modulation of energy production or consumption could occur before appreciable changes in sperm bauplan, but it remains to be established whether changes in performance (function), for example fueled by enhanced metabolism, precede changes in morphology (form), as theory would predict, because the evidence is still controversial (206, 244, 245). In addition, swimming performance is a multiparametric function in which components such as velocity and trajectory can be distinguished. Different patterns of motility, such as those seen after activation or hyperactivation, should also be distinguished in mammals. Therefore, a more careful dissection and examination of sperm kinematic parameters under appropriate conditions and characterization of sperm subpopulations, under the prism of postcopulatory selection, would be most helpful in the future. Swimming patterns are relevant because they are important determinants of male fertility, as revealed by studies in several species (246, 247).

Other sperm functions are also under selection by sperm competition. The processes that mammalian spermatozoa undergo in preparation for fertilization (capacitation and acrosomal exocytosis) are influenced by sperm competition: A comparative study using several mouse species revealed a positive association between the level of sperm competition and the proportion of cells that undergo changes compatible with capacitation or respond to a physiological agonist of acrosomal exocytosis (248), but little is so far known about the underlying molecular modifications in physiological processes that could take place in response to sperm competition. One important point to bear in mind is that these physiological processes are still poorly known and debate still exists as to how they should be framed in relation to the life of the sperm cell. For example, controversy still exists as to what constitutes capacitation of mammalian sperm and, therefore, its definition (19). In the original definition (78), the set of modifications in the female tract that prepare the sperm to engage in fertilization have been regarded as capacitation (80, 81). This definition has subsequently been challenged and perhaps needs to be refined to include only events before hyperactivation or acrosomal exocytosis (19). In addition, it has been suggested (48) that this process may not be different from other physiological interactions between sperm and female-derived factors that modify sperm behavior in other taxa, regardless of whether they engage in external or internal fertilization. Following this argument, it has been proposed that the current definition of capacitation should be entirely abandoned (48). Thus, whether the process is unique to mammals, because it includes some distinctive conditions or processes, or could be assimilated to a general phenomenon of sperm × female interactions should be examined further, using a variety of criteria.

#### 3.2.3. Common descent (phylogeny).

Another factor that may influence sperm evolution is phylogeny. Thus, sperm morphology may be similar among species because of common descent, that is, species that descend from a common ancestor, with similarities in shape and size due to this fact rather than to responses to selective forces (178, 183, 249). This is usually examined in comparative studies carried out in a phylogenetic framework to account for possible phylogenetic inertia. If such phylogenetic signals cannot be eliminated in analyses, then it is argued that it is not feasible to attribute evolutionary changes to a particular selective force. In any case, phylogeny represents more than just common descent, with the existence of developmental constraints on possible changes, particularly in form (250–252). This is, so far, a rather neglected area in the study of sperm evolution or mechanisms of sperm formation and differentiation, and perhaps more attention should be paid to this issue in the future.

BOX 1

***Spermatogonia***: Spermatogonia are located near the lamina propria in the seminiferous tubules and in contact with the Sertoli cells. In primates (e.g., monkey and man), there are three morphologically distinct subpopulations of spermatogonia: **Ad spermatogonia**, **Ap spermatogonia**, and **B spermatogonia**. In humans, Ad and Ap spermatogonia represent the undifferentiated stem cells and type B the differentiating spermatogonia. Ad spermatogonia do not divide under normal conditions of testicular function. However, Ap spermatogonia divide by mitosis to maintain their own cellular stock and, for each of them, generate two B spermatogonia. In humans, only one generation of B spermatogonia has been characterized, whereas there are four in the macaque. There are six differentiated spermatogonia in the mouse (A1, A2, A3, A4, In, and B).

***Spermatocytes***: Spermatocytes are the cells originating from B spermatogonia. They divide by meiosis. The process of meiosis involves two cell divisions, the first involves **primary spermatocytes**, and the second meiotic division, producing the haploid spermatids, involves **secondary spermatocytes**. The first meiotic division takes several days (24 days in humans); on the other hand, the second meiotic division progresses rapidly (lasting ∼5 h in humans). Primary spermatocytes are joined to each other by intercellular bridges similar to those found between spermatogonia. They are separated from adjacent Sertoli cells by distinct intercellular spaces that are modified in some regions by desmosome-like structures. The secondary spermatocytes have the haploid number of chromosomes, although their DNA content is still diploid so that when they complete meiosis the resulting spermatids have both a haploid chromosomal content and DNA content.

***Spermatids***: Spermatids are the products of the second meiotic division and lack the ability to divide. Their progressive transformation takes 23 days in humans, 22 days in rats, and 14 days in mice. It involves a morphological and functional differentiation process including formation of the acrosome, chromatin condensation, and the elimination of excess cytoplasm. There are two types of cells, **round and elongating spermatids**.

## 4. SPERM FORMATION (SPERMATOGENESIS)

### 4.1. Phases of Spermatogenesis

**FIGURE 5.**
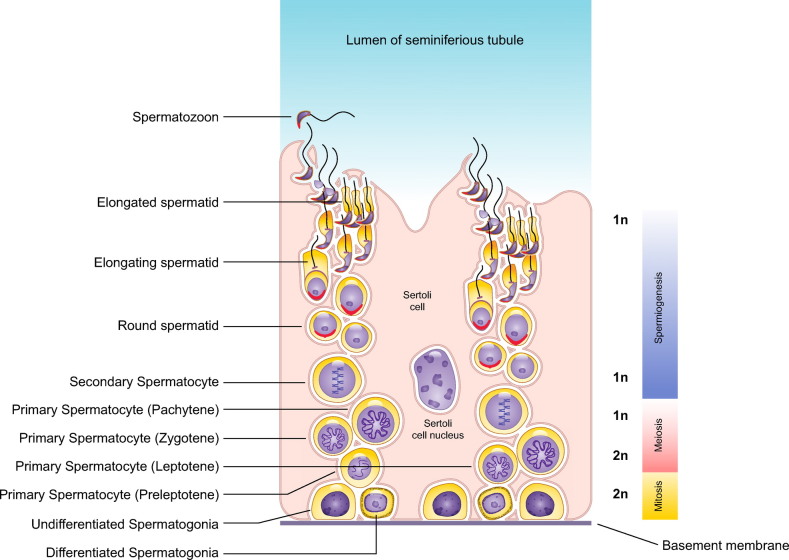
Schematic representation of the mouse spermatogenic process. Spermatogenesis advances from the base to the lumen of the seminiferous tubule in a dynamic contact with Sertoli cells. Spermatogonia reside in the basal compartment and proliferate to primary spermatocytes (preleptotene, leptotene, zygotene, and pachytene). Secondary spermatocytes undergo second meiosis and became round spermatids. The sperm differentiation phase starts at this point with morphological and molecular transformations of round spermatids into elongating spermatids. Elongated spermatids are cells in the process of spermiation undergoing the final cellular transformations. In the last steps of spermiation, most of the cytoplasm will be removed and phagocytized by Sertoli cells to form fully developed spermatozoa.

The formation of the male gamete is a highly organized process that takes place in the seminiferous epithelium. This process, known as spermatogenesis, is the sequence by which stem cells of the germ line develop into spermatozoa (BOX 1). It is usually divided into three well-characterized phases: *1*) The proliferative phase is the first phase and involves proliferation of diploid spermatogonia by mitosis. During this phase, two types of spermatogonia are produced (undifferentiated and differentiated spermatogonia). Undifferentiated spermatogonia are normally present in all stages of the seminiferous epithelium. They are responsible for the renewal of their own stock of cells and for the production of differentiated spermatogonia. Differentiated spermatogonia are cells committed to sperm production. Studies on stem cell renewal and differentiation have dramatically increased in recent years, with new advances in spermatogonia-based approaches to preserve male fertility or restore fertility in infertility cases (253–256). *2*) The meiotic phase follows the proliferative phase and involves two meiotic divisions where primary spermatocytes reduce the number of chromosomes, leading to haploid cells and genomic recombination (257). *3*) The last phase (spermiogenesis) is essential for sperm differentiation (258). During spermiogenesis, the haploid round spermatids resulting from meiosis undergo substantial structural and functional changes. During this phase there are remarkable events including the formation of new organelles such as the chromatoid body composed of RNA, the acrosome originating by fusion of vesicles, and the manchette working as a scaffold for protein trafficking and nuclear remodeling. Trafficking of proteins via acrosome-acroplaxome-manchette, nuclear condensation and remodeling, and acquisition of the species-specific shape, elimination of residual cytoplasm, assembly of the flagella, and spermiation are also hallmark events of the spermiogenic phase (FIGURE 5).

At the beginning of spermiogenesis in mammals, round spermatids have a spherical nucleus located in the center of the cell and the chromatin is decondensed (steps 1–7). Other organelles such as the acrosome and a primitive tail are developing and experiencing transformation during these steps. The acrosome is assembled from trans-Golgi and extra-Golgi vesicle pathways, and then it spreads, covering the apical part of the nucleus. Simultaneous with acrosome assembly, flagellum formation starts with docking of the centrosome to the nuclear membrane at the opposite side of the nucleus. The axoneme then extends from the proximal centriole. As the cell differentiation process advances, the nucleus moves toward one pole of the cell (step 8 in rat and mouse). Then, the cells start to mold an asymmetric nucleus supported by the manchette scaffold until step 14. Assembly of accessory structures in the tail develops after axoneme initiation. Fibrous sheath longitudinal columns begin to appear around the distal end of the axoneme and then extend “backwards” as the axoneme extends. The transversal ribs are assembled during steps 11–15 in the rat, and outer dense fibers start simultaneously with manchette formation. The mitochondrial sheath is the final structure of the flagellum to be assembled. Chromatin condensation begins around steps 12 and 13. In nonrodent species with spatulated sperm head, the nucleus lacks dorso-ventral asymmetry and the characteristic hook, but the other events remain similar. For these dramatic changes to happen, several mechanisms have been proposed. A recent excellent review (259) deals with cellular and molecular aspects of spermiogenesis that are not covered in detail here.

The underlying genetic control of spermatogenesis is complex and not well understood. Increasing evidence supports the idea that transcriptional mechanisms are quite different in haploid germ cells with respect to somatic cells (260). These differences include the use of distinct promoter elements and specific transcription factors as well as mechanistically different routes for activation (261). Widespread transcription in the testis has been shown to involve >80% of all genes in humans as well as in other species. Recently, a transcriptome analytic study has characterized the dynamics of gene expression in germ cells during the course of spermatogenesis (262). This study has shown that a total of 108 genes are uniquely expressed in pachytene spermatocytes and their expression is not carried over to round spermatids or sperm. Moreover, 323 genes are exclusively expressed in round spermatids, and 178 are only present in sperm. Interestingly, six genes expressed in spermatocytes are lost in spermatids but reappear in sperm. Many of the proteins synthesized during spermatogenesis are transcribed from the haploid nucleus and/or are translated from stored mRNAs. A comparative sperm proteomic analysis in three closely related mouse species has revealed significant differences between species in proteins involved in fertilization, including those that govern axoneme components and metabolic proteins (263). In addition, proteins that may underlie the diversification of spermatozoa were more likely to experience translational repression, suggesting that composition of spermatozoa is affected by the evolution of mechanisms that control translation. Because these species vary in their levels of sperm competition, it was possible to identify classes of proteins that are functionally coherent in relation to differences in postcopulatory sexual selection. Moreover, relaxed or intensified levels of sperm competition seemed to impact on the molecular composition of sperm cells (263). More recently, it has been proposed that widespread testis transcription facilitates germline DNA repair and ultimately modulates gene evolution rates (264). It was found that genes expressed during spermatogenesis have lower rates of nucleotide mutations compared with unexpressed genes. Moreover, the increased transcription during spermatogenesis may facilitate a transcriptional scanning process that systematically detects and repairs damage in the DNA by transcription-coupled repair. Interestingly, 90% of all protein-coding genes in germ cells are expressed. In contrast, somatic cells in the testis express only 60% of the genes, of which >99% overlap with the genes expressed in germ cells (264).

### 4.2. Control of Spermatogenesis

Although it is generally considered that spermatogenesis is species specific, with regard both to the morphology of the sperm cell generated after differentiation and to the timing required to generate the mature sperm cell, there are a series of conserved mechanisms. Moreover, it is possible that early stages of spermatogenesis involving proliferation of spermatogonia may bear a degree of similarity among species, whereas the latter stages of spermatid differentiation may be more divergent, as they involve processes that end up in species-specific sperm cell morphologies. The characteristics of spermatogonial stem cells and dynamics of spermatogonial proliferation, along with regulatory mechanisms (253, 255), as well as those corresponding to the meiotic phase (265), have received much attention and are not covered here. Instead, we focus on aspects of sperm differentiation and control of sperm remodeling during the last phase of spermatogenesis. Thus, there is the question of what controls the morphology and timing of sperm differentiation and remodeling. Evidence suggests that the germ cell, and not the Sertoli cell, is the cell that primarily defines the remodeling of spermatids. We first review the roles of Sertoli cells and then summarize work on heterologous (xenogeneic) spermatogonia transplantation that has led to the conclusion that germ cells themselves are in control of spermatid differentiation and remodeling.

Sertoli cells play fundamental roles in spermatogenesis because they *1*) maintain the blood-testis barrier, which creates the appropriate environment for the occurrence of spermatogenesis; *2*) provide physical support for the germ cells; *3*) serve as “nurse cells” because of their involvement in the nutritional support of the male germ cells; *4*) control hormonal regulation; *5*) carry out the phagocytosis of apoptotic germ cells and residual bodies; and *6*) release the spermatids toward the tubular lumen during spermiation (266). Some studies have also shown that the number of germ cells supported by a single Sertoli cell is limited and depends on the species (267, 268). However, it is unclear how much of the process of germ cell differentiation is driven by these cells.

Spermatogonia can be transferred from an infertile male mouse to a fertile one in which the donor spermatogonial stem cells establish spermatogenesis and produce spermatozoa that transmit the donor haplotype to progeny (269, 270). This observation prompted studies of spermatogonia transplantation between species to examine whether the Sertoli or germ cells control the process of spermatogenesis. Marked testis cells from transgenic rats transplanted to the testes of immunodeficient mice colonized mouse seminiferous tubules and subsequently exhibited continuous spermatogenesis generating spermatozoa with a head and flagellum morphology characteristic of the rat (269, 271). Interestingly, although rat Sertoli cells were transplanted with rat donor germ cells, they were absent in seminiferous tubules colonized by the rat germ cells (272, 273). This suggests flexibility in the supporting role of the Sertoli cell in species that diverged a long time ago. Because rat spermatogenesis has a cycle length that is 50% longer than that of mouse spermatogenesis (rat: 52 days, mouse: 35 days), the question that also emerged was whether, in the mouse, rat spermatogenesis (if influenced by mouse Sertoli cells) had a shorter cycle. It was found that the germ cell genotype is the one that controls the cell cycle during spermatogenesis. Furthermore, both rat and mouse spermatogenesis developed concomitantly in the same mouse host after transplantation. Under these conditions, two different timing regimens for germ cell development were observed in the recipient mouse testis: one of rat and one of mouse duration (272). Thus, rat germ cells that were supported by mouse Sertoli cells always differentiated with a timing that was characteristic of the rat (52 days) and generated the spermatogenic structural pattern of the rat. These results thus suggest that the cell differentiation process of spermatogenesis is regulated by germ cells alone.

To examine whether sperm generated after xenogeneic spermatogonia transplantation are functional, rat spermatozoa produced in mouse testis were recovered from seminiferous tubules and microinjected into rat oocytes, which were then transferred to recipient females. Rat offspring were born from donor spermatogonial cells, and such offspring were fertile and had a normal imprinting pattern (274). The reciprocal xenogeneic transplantation was also successful (275, 276).

Hamster testis cells transplanted into a testis of an immunodeficient mouse also underwent spermatogenesis (277), despite a bigger difference between hamster and mouse sperm morphology than between rat and mouse sperm cells and older divergence times in the former pair (>16 million yr) in comparison to the latter (∼11 million yr). In this case, abnormalities were observed in hamster spermatids in seminiferous tubules of recipient mice. Hamster spermatozoa were seen in the epididymis of recipient mice, but most spermatozoa lacked acrosomes, and heads and tails were separated. Defects in hamster spermatogenesis occurring in mouse seminiferous tubules could be due to lower compatibility and a limited capacity of mouse Sertoli cells to support the much larger hamster spermatozoa, which also have a different head shape (277).

Transplantation of germ cells from other species (rabbit, dog, pig, bull, and stallion) into mouse testes was also examined (278–280). These combinations represent an increased phylogenetic distance in comparison to the previous rat-mouse or hamster-mouse transplantations. Germ cells from rabbits or dogs colonized the mouse testis but did not proliferate or differentiate beyond the stage of spermatogonial expansion (279). Pig donor germ cells formed chains and networks of round cells connected by intercellular bridges, but later stages of donor-derived spermatogenesis were not found (278, 280). Bull testis cells developed predominantly into fibrous tissue. Few stallion germ cells proliferated in mouse testes (280). Thus, germ cells from domestic animals do colonize the mouse testis but do not differentiate beyond the stage of spermatogonial expansion.

Among primates, baboon spermatogonial stem cells readily established germ cell colonies in recipient mice and survived for a period of ∼6 mo, but differentiation into spermatozoa did not take place (281). After xenotransplantation of human testis cells to mouse seminiferous tubules, no donor germ cells were identified in recipient testes (282). In contrast, another study (283, 284) claimed that transplantation of human testis cells to mouse and rat seminiferous tubules led to the production of spermatozoa in >25% of recipients. Subsequent studies (285) found that a high proportion of mouse recipient testes were colonized by human testis cells obtained from several patients. Human spermatogonial stem cells survived in mouse testes for at least 6 mo and proliferated during the first month after transplantation. However, no differentiating human spermatogonia were identified, and meiotic differentiation did not take place in mouse testes.

The fact that rabbit, baboon, human, pig, bull, or dog spermatogonial stem cells and undifferentiated spermatogonia survive in mouse seminiferous tubules for a long period after transplantation suggests that antigens, growth factors, and signaling molecules that are required for interaction of these cells and the testis environment are highly conserved (281). Because these species diverged from rodents ∼75–85 million yr ago (286), it seems that there could be a high degree of conservation in the first stages of spermatogenesis. On the other hand, because differentiation of germ cells does not take place in mouse seminiferous tubules when spermatogonia from species other than rat or hamster are transplanted, molecules necessary for stages of spermiogenesis appear to have undergone great divergence between rodents and rabbits, primates, ungulates, or carnivores. Support for this idea comes from studies of xenografting in which testicular tissue fragments were transplanted subcutaneously to immunodeficient mice (253, 287). Under these conditions communication between the endocrine system and the donor testis become functional, and this leads to full spermatogenesis and competent sperm cells in grafts from species that are distantly related to mice, such as primates (288, 289), carnivores (290, 291), and ungulates (292–296).

In addition to the fundamental questions related to the regulation of spermatogenesis, spermatogonia transplantation has potential applications for animal transgenesis (297), studies of human infertility, domestic animal production, or animal conservation (253). The possibility of using cryopreserved testis cells (rats: Refs. 269, 274; hamsters: Ref. 277) represents an opportunity to store material from valuable animals and use them when suitable recipients are identified or culture techniques are developed. The question of whether xenogeneic germ cell transplantation could be achieved with other combinations of species is relevant, but the distance between host and the species of interest is still potentially a problem. In attempts to use xenogeneic germ cell transplantation for species conservation, procedures were explored using the domestic cat as a recipient for the preservation and propagation of male germ plasm from wild felids (298) Both syngeneic and xenogeneic transplants revealed that spermatogonial stem cells were able to colonize and differentiate successfully in the recipient cat testis, generating elongated spermatids several weeks after transplantation. After >3 mo of transplantation, ocelot spermatozoa were observed in the cat seminiferous tubules and in the epididymis; the morphology of ocelot spermatozoa differentiating under these conditions appeared normal, and it seems that they could be differentiated by shape and size (298). At the present time, it seems that the domestic cat may be suitable as a recipient for other wild felids because no signs of immunorejection were observed (298). It remains to be established whether these spermatozoa are functionally competent by examination of their fertilizing ability.

In summary, the studies mentioned above support the hypothesis that the germ cell seems to drive its own process of morphological change during cell differentiation. Two main factors have been proposed to exist in the germ cells to influence such morphological changes: *1*) external forces applied to the spermatid nucleus either by cytoplasmic microtubules or by ectoplasmic filaments from Sertoli cells (299) and *2*) internal nuclear forces resulting from a controlled pattern of aggregation of DNA and proteins during the condensation of the chromatin (35). These mechanisms are discussed in detail in sect. 5.

### 4.3. Energetics of Sperm Formation

A considerable amount of energy is directed toward reproductive functions, in searching for mates, production of spermatozoa, or both. It is well known that sperm cells are costly (300) and that germ cells have important energy demands. Evidence for substantial costs of spermatogenesis comes from *Drosophila*, in which increasing sperm length delays male reproductive maturity (301), increases resources required for sperm production and packaging (302, 303), and selects for prudent male sperm-production strategies (304).

Sertoli cells provide nutritional support for male germ cells during the differentiation phase, particularly to fulfill their energetic needs (305). The metabolic requirements of Sertoli cells and germinal cells are quite different. Sertoli cells have been shown to produce lactate and pyruvate. Three-quarters of this production is made to satisfy germ cells, whereas β-oxidation is suggested to be used only for the Sertoli cells’ own energy needs (305). Germ cells have different metabolic requirements during their differentiation states. The spermatogonia are spatially located near blood sources and can rely on glucose as a source of ATP via glycolysis (306). Spermatocytes and spermatids reside in the luminal compartment of the testis and can be at some distance from the blood supply. These cells may use glycolysis to generate ATP and possibly also use lactate as a substrate (307–309). Spermatids have the enzymes that make up the glycolytic pathway, but glucose metabolism alone does not seem capable of maintaining ATP levels, because in the absence of other energy sustrates spermatids experience depletion of ATP (310). Thus, they need pyruvate and lactate for survival and likely rely on the supply of lactate from the Sertoli cells. Several genes encoding sperm-specific forms of enzymes of the glycolytic pathway are expressed only in spermatids (306, 307). There is also selective expression of lactate dehydrogenase in advanced germ cells in the adluminal compartment and, since lactate dehydrogenase C (LDHC) converts lactate into pyruvate and pyruvate can be converted to acetyl coenzyme A, which would fuel OXPHOS, postmeiotic cells may rely more on mitochondrial OXPHOS activity (309). 

The mitochondria in germ cells change during spermatogenesis (309). Spermatogonia and early spermatocytes have mitochondria that are similar to those in somatic cells, whereas late spermatocytes, spermatids, and spermatozoa have more condensed and efficient mitochondria (311, 312). During the final stages of spermiogenesis mitochondria are lost in residual bodies. For example, <100 mitochondria remain in the mouse sperm cell, and they rearrange in tubular structures that are anchored helically around the nine outer dense fibers and the axoneme in the sperm midpiece (313, 314). As a consequence, the amounts of mitochondrial proteins and DNA molecules are also reduced (315, 316).

### 4.4. Evolution of Spermatogenesis

The overall process of spermatogenesis appears to be conserved among many taxa (255), although several characteristics, such as sperm morphology and timing of the differentiation process from spermatogonia to mature spermatozoa, are unique and species specific. In mammalian species, the time required for the completion of spermatogenesis is unique and of a fixed duration for each species. It has generally been assumed that cell-cell interaction between germ cells and Sertoli cells could be a limiting factor in the production of spermatozoa (317) and that the duration of cell cycles and cellular organization may be the result of this interaction. Males appear to have the plasticity to modify the production of spermatozoa and generate more sperm, or to adjust sperm morphology, in response to social conditions such as the perceived risk of sperm competition (318). Under conditions of sperm competition, males would benefit if they could transfer a higher number of spermatozoa and/or sperm with a morphology that gives them an advantage over rival males (319, 320).

Males could potentially modify sperm output by increasing the relative size of testes, by modifying testis architecture, by making production more efficient, or by changing the speed at which sperm are produced. In birds and mammals subject to high sperm competition levels, the proportion of spermatogenic tissue contained within the testis increases (321–324). Species under strong sperm competition generate more round spermatids per spermatogonium and have Sertoli cells that support a greater number of germ cells, both of which are likely to increase the maximum sperm output (325). High levels of sperm competition lead to shorter length of spermatogenic cycles in mammals, which indicates faster rates of spermatogenesis (322, 326).

In a comprehensive comparative analysis of almost 100 mammalian species, changes in testis size, testicular architecture, kinetics of spermatogenesis, and sperm reserves were examined in relation to sperm competition in an integrative manner (321). It was found that higher levels of sperm competition correlated with higher proportions of seminiferous tubules, shorter seminiferous epithelium cycle lengths (SECLs), which reduce the time required to produce sperm, and higher efficiencies of Sertoli cells involved in sperm maturation. These responses to sperm competition were found to result in higher daily sperm production, more sperm stored in the epididymides, and more sperm in the ejaculate (321). Although there was a strong relationship between SECL and the duration of spermiogenesis, the levels of sperm competition were not associated with the latter (i.e., the period of sperm differentiation) (321).

**FIGURE 6.**
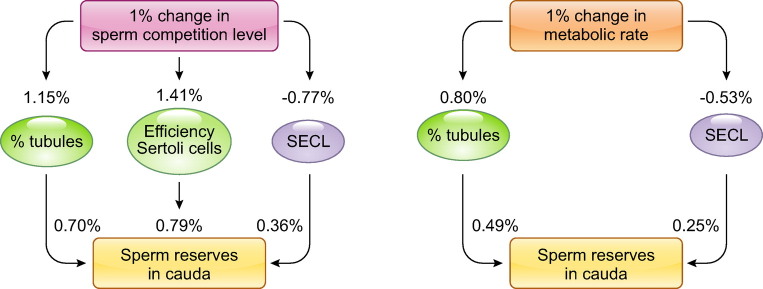
Relationships between sperm competition, or metabolic rate, and testis architecture, kinetics of sperm formation, and sperm numbers in mammals. The schematic representations show dynamic variations in testicular parameters, or sperm reserves in the epididymis, in relation to sperm competition or mass-specific metabolic rates. In the model, level 1 (*top*) corresponds to sperm competition (*left*) or metabolic rate (*right*), level 2 (*middle*) corresponds to testicular parameters [% tubules, efficiency of Sertoli cells, seminiferous epithelium cycle length (SECL)], and level 3 (*bottom*) corresponds to sperm reserves in caudae epididymides. Numbers at the end of each arrow between levels 1 and 2 are the relative variation in the dependent variable (level 2; testicular parameters) caused by a variation of 1% of the independent variable (level 1; sperm competition or metabolic rate). Numbers at the end of each arrow between level 2 and 3 variables indicate the relative variation in the dependent variable (level 3; sperm reserves) caused by the change in the variable (level 2) due to a 1% increment in level 1. Percentages of relative variation were calculated using the slopes and intercepts estimated by phylogenetic generalized least squares (PGLS) models (321). % tubules, % of the testicular tissue occupied by seminiferous tubules; sperm reserves in cauda, number of spermatozoa in the caudae epididymides.

A dynamic model considering all the information available for mammalian species was used to jointly analyze the data in order to understand the relative contribution of variations in testis architecture and kinetics of spermatogenesis in response to sperm competition (FIGURE 6). Three hierarchic levels of variables were identified based on the hypothetic relationships tested in the models: *1*) sperm competition; *2*) SECL, efficiency of Sertoli cells, and percentage of the testis occupied by seminiferous tubules (% of tubules); and *3*) number of sperm in the caudae epididymides (sperm reserves). The slopes and intercepts estimated by phylogenetic generalized least squares (PGLS) models were used to predict the relative influence of one level on the successive levels. Dynamic variations of the three levels were assessed by first examining a 1% variation in the range of the predictor (level 1) on changes in the dependent variable of the next level (level 2). Then, for the relationships between levels 2 and 3, the variation introduced in the predictor variable at level 2 was that resulting from the variation of 1% in the range in the level 1 predictor. For example, an increase in sperm competition equivalent to 1% of sperm competition range produced an increase of 1.15% of the variable range of seminiferous tubules; such 1.15% increase in tubules resulted in 0.70% increase in sperm numbers in the cauda (321). Results clearly revealed a higher impact of sperm competition on sperm architecture than on sperm kinetics, in turn resulting in a higher effect of the former on sperm reserves in the cauda epididymis (FIGURE 6). The highest effect of sperm competition was seen on the efficiency of the Sertoli cells, which, altogether, had the highest effect on sperm reserves.

The effect of metabolic rate on testicular traits and sperm reserves was also examined because low mass-specific metabolic rate, as seen in large-bodied species, may limit processing energy and resources efficiently enough at both the organismic and cellular levels. In the same dynamic model explained above, changes in metabolic rate had a higher impact on sperm architecture than on sperm kinetics, with a higher effect of testicular architecture on sperm reserves (FIGURE 6). Furthermore, the two variables that require processing resources at faster rates (SECL and efficiency of Sertoli cells) only responded to sperm competition in species with high mass-specific metabolic rate. Thus, increases in sperm production with intense sperm competition take place via a complex network of mechanisms, with some being constrained by metabolic rate (321).

## 5. MOLECULAR MECHANISMS UNDERLYING SPERM FORMATION

**FIGURE 7.**
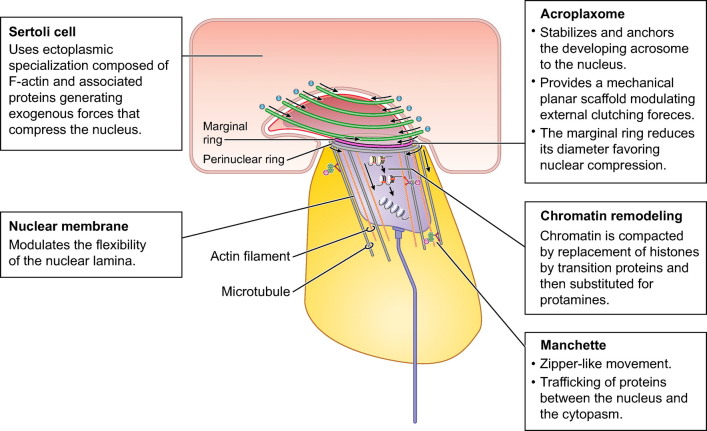
Schematic representation of the processes involved in sperm head shaping during spermiogenesis. The ectoplasmic specialization in Sertoli cells encircles >1/3 of the spermatid head and generates external pressure. The acroplaxome, also covering >1/3 of the head, modulates the external forces and anchors the acrosome to the nucleus. The manchette, a scaffolding of microtubules and actin filaments, encircles the 2/3 remaining area of the nucleus. It connects intimately with the nuclear lamina and generates internal forces by a zipperlike movement. Additionally, the manchette provides tracks for the trafficking of proteins. The nuclear membrane restructures its molecular composition, redistributing proteins to adjust the flexibility of the membrane to allow for external and internal forces. Replacement of histones by transition proteins and then protamines makes the last twists for chromatin condensation.

One of the most remarkable cellular changes accompanying sperm differentiation is the remodeling of head architecture (327). As described in sect. 3, there is a rich variety of shapes of sperm heads. Globular forms are common among fish. Amphibia have filiform, cylindrical, tapering, or long heads. Flattened ovoid heads are predominant in mammals, although murine and cricetid rodents generally have a falciform, sickle-shaped head. We review in this section the mechanisms that influence the remodeling of the sperm head (FIGURE 7). Most of the current knowledge comes from several studies performed in mammals, and little is known for other species or from an evolutionary point of view. Whenever possible, comparison of processes occurring in different species is attempted.

### 5.1. Role of Sertoli Cells

**FIGURE 8.**
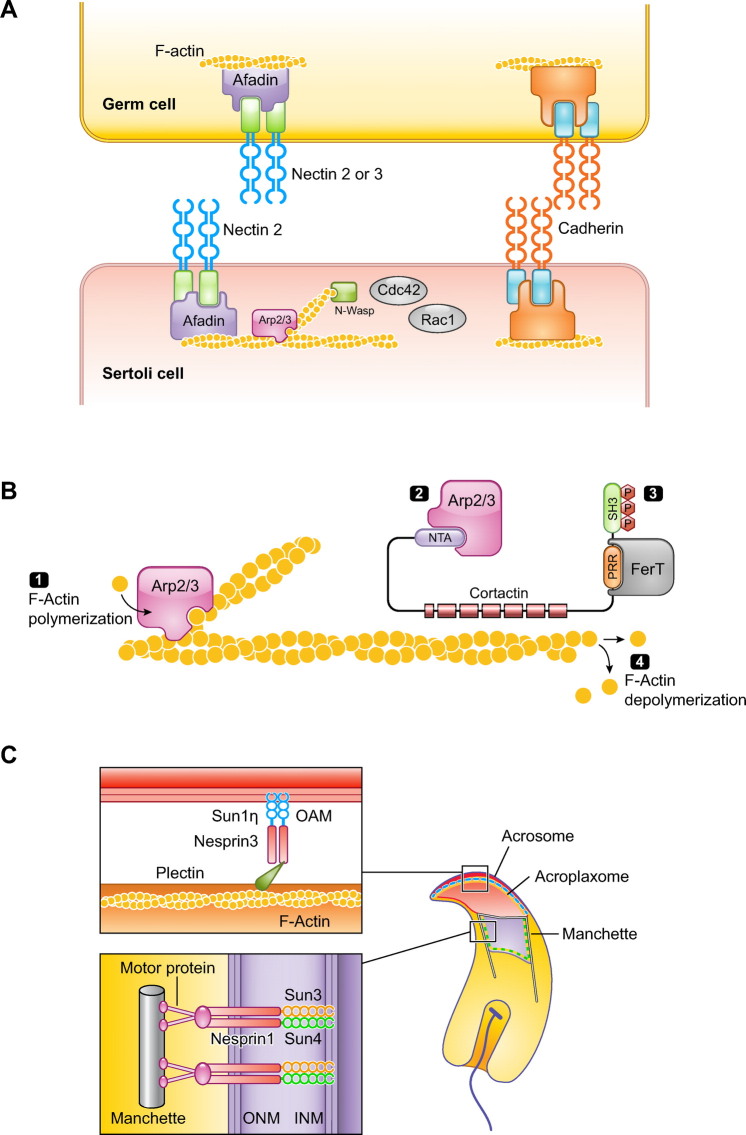
Molecules involved in the spermatid head remodeling. *A*: a molecular model that illustrates the structural association between Sertoli cells and spermatids during head remodeling at the site of ectoplasmic specialization. *B*: diagram illustrating the binding of Cortactin to F-actin. *1*: Arp2/3 complex promoting F-actin polymerization. *2*: Arp2/3 regulating Cortactin function. *3*: FerT mediating tyrosine phosphorylation of Cortactin. *4*: Phosphorylation of Cortactin promotes F-actin depolymerization. *C*: schematic representation of the distribution of SUN domain proteins during nuclear elongation. Yellow, Sun3; blue, Sun1 and Sun1η; green, Sun4. Posterior and anterior spermiogenesis-specific linker of nucleoskeleton and cytoskeleton (LINC) complexes are shown in *insets*. INM, inner nuclear membrane; OAM, outer acrosomal membrane; ONM, outer nuclear membrane.

The Sertoli cells are specialized cells that surround the germinal cells. Among their numerous functions, orchestrating germ cell development and differentiation, they are believed to also participate in the compression and remodeling of the sperm head via mechanical forces. During spermatid elongation (step 8), the Sertoli cells spread over more than one-third of the sperm head and thus contribute to nuclear and head reshaping (328). However, the underlying mechanisms are not well understood. The Sertoli cells display an ectoplasmic specialization (ES) that participates in the remodeling of the sperm head, and this structure is thought to be conserved in mammals. However, it is not clear how this structure would influence the formation of oval or falciform sperm heads. At the molecular level, the ES is formed by a layer of actin bundles packed in parallel hexagonal arrays and surrounded by cisternae of endoplasmic reticulum (295). Associated proteins including afadin and nectin 2 and 3 are also part of this structure (329). Nectin 2- and 3-deficient mice show severe disorganization in the sites where Sertoli cells contact with spermatids, resulting in aberrant spermiogenesis and sterility (330, 331). The actin-related protein (Arp) 2/3 complex has been localized to the ES and may contribute to the formation of the actin network at the site. This complex includes Arp3, N-Wasp, cortactin, and regulators such as Rac1 and Cdc42 (332). F-actin bundles present in the ES are believed to be noncontractile. It has been proposed that the mechanical forces may be driven by dynamic changes in polymerization and depolymerization of F-actin assisted by the Arp2/3 complex (333) and Fer kinase (334) (FIGURE 8*A*). It is not yet clear how these proteins interact with F-actin and what the molecular mechanisms behind the role of Sertoli cells in the remodeling of the head are. Thus, this is an important area that needs further investigation.

### 5.2. The Acroplaxome

The acroplaxome was described for the first time in rat spermatids by Kierszenbaum and collaborators (335) as a cytoskeletal plate linking the inner acrosomal membrane to the nuclear envelope. At the molecular level, the acroplaxome is composed of F-actin, keratin 5 (335), and myosin Va (336). This structure also includes a specialized electron-dense material named marginal ring (336), which is made up of keratin 5-containing intermediate filaments inserted into a plaque associated with the leading edge of the inner acrosomal membrane. The array of F-actin is dynamically modulated in the acroplaxome by Arp2/3 complex (333), tyrosine kinases targeting cortactin (337), as well as FerT (338) (FIGURE 8*B*). The testis-specific proteins profilin-3 (PFN3) and profilin-4 (PFN4), crucial for actin microfilament dynamics, were also reported to localize to the acroplaxome (339). It has been proposed that the acroplaxome plays a role in stabilizing and anchoring the developing acrosome to the nucleus of elongating spermatids. Additionally, it may also provide a mechanical planar scaffold modulating external clutching forces generated by the ectoplasmic specialization of Sertoli cells encircling the elongating spermatid nucleus (335). Mutant mouse models in which spermatid nuclear shaping was defective provided clues concerning the significance of the acroplaxome in the elongation of the spermatid nucleus. In the *azh* mutant mouse, a relatively large number of spermatids display abnormally shaped nuclei and spermatids in which the acroplaxome-containing region is particularly indented (336, 340). *Hrb* mutant males are infertile, and both spermatids and sperm are round headed and lack the acrosome. Although the acroplaxome plate is present in the mutant spermatids, its composition is affected, since it is deficient in keratin 5 but not F-actin (329). Additional proteins were shown to localize to the acroplaxome of rat spermatids, including the ubiquitin protein ligase Rnf19a, the component of the 26S proteasome Psmc3 (341), the outer dense fiber (ODF2) protein (342), the Golgi protein GMAP210, and the intraflagellar protein IFT88 (343). Recently, lamin A/C has been proposed to be part of the acroplaxome in the mouse (344). The decreased expression of lamin A/C following injections of siRNA against the *Lmna* gene causes significant alterations in nuclear elongation and acrosome formation (344). However, further studies are required to understand the role of lamin A/C in the acroplaxome (327). Another protein reported to be present in the acroplaxome is MARCH7, an E3 ubiquitin ligase important for protein degradation (345). Although confocal images show colocalization with β-actin in the head of elongating spermatids, it is difficult to conclude that MARCH7 is a component of the acroplaxome with the experiments performed by Zhao and collaborators (345). Superresolution or electron microscopy studies may provide better clues for the acroplaxome localization of this protein. Unfortunately, since the discovery of the acroplaxome (335), very few advances have been made in the characterization of this structure and the molecular mechanisms that take place here. Moreover, it is not clear whether this structure and its function are conserved among species. Therefore, this is an area of research that deserves further investigation. The combination of state-of-the-art superresolution microscopy and cryo-electron microscopy may bring about valuable discoveries in this area.

### 5.3. The Manchette

The manchette is a transient perinuclear organelle that is also believed to participate in the elongation of the sperm head and nucleus. The timing of the development of the manchette is very precise. In the mouse and the rat, it assembles around step 8 during spermatid elongation and disappears when the elongation and condensation of the nucleus are nearly completed (step 14).

This skirtlike organelle consists of bundles of microtubules connected to a perinuclear ring and filaments of actin intercalated between the microtubules (17, 346) (FIGURE 7). Endoplasmic reticulum is aligned along the cytoplasmic face of the manchette. The manchette microtubules appear to emanate from the perinuclear ring at the base of the acrosome. However, there is still controversy about whether the microtubules are nucleated in the perinuclear ring or are nucleated elsewhere and are later linked to this ring. Two specific nucleation sites for the manchette microtubules have been postulated: the perinuclear ring and the centrosome. However, detection of plus-end tracking proteins (EB3 and CLIP-170) together with the absence of γ-tubulin disagree with the idea of the perinuclear ring as the microtubule nucleation site (346–348). Instead, supporting evidence indicates that the centriolar adjunct serves as a nucleator of manchette microtubules with their plus ends reaching toward the perinuclear ring (348, 349). Another theory proposed multiple microtubule organizing centers that organize from existing cytoskeletal microtubules (328). Further experimental studies should be performed to bring light to this issue.

The importance of the manchette in sperm head shaping has been a matter of debate because of discrepancies found in different species (35). In a number of species the manchette microtubules are absent during spermiogenesis (e.g., scorpion, Anthozoa, Chaetognatha, crested tinamou) (350–353) or disappear before profound changes in nuclear shape (35, 354). Species such as the ostrich display a circular and longitudinal manchette around the nucleus (280). However, with the help of murine mutant models it has become evident that the manchette plays an important role in head shaping during spermatid differentiation in mammals. In general, the spermatid nucleus assumes a parallel shape to the manchette, and when the manchette is absent the nucleus adopts a round shape as seen in models with caudally displaced manchette and in mutants that lack nucleus-manchette connection (reviewed in Ref. 348). During spermatid elongation, the marginal ring of the acroplaxome and the perinuclear ring of the manchette reduce their diameter as they gradually descend along the nucleus toward the spermatid tail (329). This zipperlike movement helps with nuclear condensation and shaping (348). In the case of rodents with hook-shaped heads, this zipperlike movement of the manchette generates forces that help to create the dorsal and ventral nuclear surfaces (355). Yet the molecular mechanisms driving this force are not well understood. Many molecules have been found to anchor or harbor around the manchette (17). They assist with nuclear condensation, spermatid differentiation, and tail formation (348). However, their molecular functions are largely uncharacterized. Recently, in silico analysis has drafted a putative interacting protein network that represents a framework for future investigations (17). Several proteins have also been shown to localize to the manchette, and their mutation in murine models resulted in perturbations in the manchette and alterations in the nucleus and head shape **(**TABLE 2**)**. Bay way of illustration, SPAG17, a protein originally described as a microtubule central pair protein present in the flagellar axoneme (401), has been found to localize to the manchette and shown to be essential for nuclear shaping and chromatin condensation (390), a role that extends beyond its proposed role in flagellar function. SPAG17 is a poorly understood protein, and the molecular mechanisms behind its pleiotropic functions are unknown (401). Nevertheless, it appears to be associated with trafficking of proteins during spermatid differentiation (390). The coiled-coil domain containing 42 (CCDC42) protein has been found to be enriched in the perinuclear ring and to colocalize with acetylated tubulin in the manchette (358). A *Ccdc42*-mutant mouse displays defects in the nuclear morphology of elongating spermatids (357). The molecular function of CCDC42 is unknown, but it was shown to interact with ODF1 and ODF2 (358).

**Table 2.** Knockout mouse models affecting the nuclear shape and the manchette during sperm differentiation

Gene	Protein	Spermiogenesis Phenotype	Interaction	References
*Azi1/CEP131*	CEP131	Short tail, disorganized sperm tail structures, ectopic and elongated manchette	BBS4	(356)
*Ccdc42*	CCDC42	Abnormal shaped heads, detached acrosome-acroplaxome, lack of flagella	ODF1, ODF2	(357, 358)
*Cfap43*	CFAP43	Abnormal manchette, disorganized ectoplasmic specialization, and sperm head and flagella deformities		(359)
*Clip170*	CLIP170	Abnormal formation and maintenance of the manchette and abnormally shaped sperm heads	LIS1, UBE2	(17, 360)
*Cntrob*	CNTROB	Ectopic and asymmetric perinuclear ring and manchette, detached centrosome, decapitated and disorganized tails	KRT5, tubulin	(361)
*Dlec1*	DLEC1	Deformed head, shortened tail, and abnormal manchette organization	α/β-Tubulin, TCP-1, BBS2, BBS4, BBS5, BBS6	(362)
*Drc7/Ccdc135*	DRC7	Elongated manchette and sperm with shorter tails and abnormal head shapes	DRC5	(363)
*E-Map-115*	E-MAP-115	Ectopic manchette along regions of the nucleus that normally do not display manchette and tail appears normal	Kinesin 1	(364)
*Fam46c*	FAM46C	Headless spermatozoa due to defects in the connecting piece		(365)
*Fused/Axin1*	FU	Periaxonemal abnormalities, manchette elongated and malformed, acroplaxome affected	KIF27/ODF1	(366)
*Gopc*	GOPC	Lack of the acrosome, lack of postacrosomal sheath and the posterior ectopic and misplaced manchette; impaired mitochondrial sheath assembly in the epididymal spermatozoa, coiled flagella	Golgi-160, RAB6A, GRID2, BECN1, RHOQ, ACCN3, CFTR, CSPG5.	(367, 368)
*Hook1/azh*	HOOK1	Elongated and disorganized manchette microtubules, knoblike shape of the head, weak head-tail connection, and bending of the tail	RIMBP3, CCDC181	(335, 369–371)
*Ift20*	IFT20	Abnormal head shape, elongated manchette, disorganized tail components	SPEF2, GMAP210, COPS5, IFT88, IFT172	(17, 372)
*Ift81*	IFT81	Abnormally shaped heads and lack of tails		(373)
*Ift88*	IFT88	No axoneme, disorganized tail components, malformed HTCA, ectopic perinuclear ring and manchette elongated	GMAP210	(343)
*Ift172*	IFT172	Abnormal nuclear and acrosome morphology, elongated manchette, abnormal axoneme accessory structures, multiple centrosomes	IFT20, IFT27, IFT88	(17, 374)
*Iqcg*	IQCG	Short tail and disorganized sperm tail structures, irregular nucleus and localized to the manchette	Calmodulin	(375)
*Katnal2*	KATNAL2	Abnormal nuclear morphology, detached acrosome, elongated manchette, absence of sperm tail	KATNB1, δ- and ε-tubulin	(376)
*Katnb1*	KATNB1	Sperm tail mobility affected, manchette elongated and knoblike	Katanin 60	(377)
*Kifnb1*	KIF3A	No axoneme, disorganized tail components, manchette elongated and knoblike shape of the head	KIF3B, KAP, MNS1, KBP.	(378)
*Lrguk1*	LRGUK1	Short tail, acrosome acroplaxome detached, manchette MTs unevenly distributed and elongated manchette	HOOK2	(379)
*Meig1*	MEIG1	Disorganized sperm tail structures, disrupted manchette structure reported, and round or detached heads	PACRG, SPAG16	(380–382)
*Pacrg*	PACRG	Disorganized sperm tail structures, disrupted manchette structure reported, and round or detached heads	MEIG1	(383)
*Rim-bp3*	RIMBP3	Abnormalities in sperm heads characterized by deformed nuclei and detached acrosomes	HOOK1	(384)
*Sept12*	SEPT12	Defective sperm heads, bent tails, premature chromosomal condensation, and nuclear damage	CDC42	(385, 386)
*Spag6*	SPAG6	Abnormal spermatid head, midpiece fragmentation, truncated flagella, decapitated sperm	Snapin, COPS5, SINK2, TCTE3, SPAG16L, TAC1, Moesin, BBS4, DAZL, ACTR2, MGP SPAG17	(387–389)
*Spag17*	SPAG17	Abnormal nuclear morphology and chromatin condensation, detached acrosome, elongated manchette, absence of mature sperm	SPAG6, SPAG16	(389, 390)
*Spef2*	SPEF2	Short tail, elongated manchette, and disorganized sperm tail structures	IFT20	(391, 392)
*Spem1*	SPEM1	Head bent back, midpiece wrapped around head and retained cytoplasm	RANBP17, UBQLN1	(393, 394)
*Stk33*	STK33	Oversized acrosomal tips, bifurcated heads, elongated manchette, and disorganized tail structures	TUBA1B, ACTN4	(395)
*Sun3*	SUN3	Abnormalities in the manchette and sperm heads characterized by deformed nuclei and missing, mislocalized, or fragmented acrosome, as well as multiple defects in sperm flagella	SUN4	(396)
*Sun4*	SUN4/SPAG4	Round-headed sperm, abnormal acrosome, severely disorganized manchette, and coiled tails	SUN3, Nesprin1, ODF1	(397, 398)
*Ube2b*	UBE2B	Mislocation of the longitudinal columns of the FS, head shape and MS abnormalities, acrosomal defects, and ectopic manchette	RAD18	(399, 400)

FS, fibrous sheath; HTCA, head-to-tail coupling apparatus; MS, mitochondrial sheath; MT, microtubule.

Depending on the species, the manchette is in intimate contact with the nucleus. It connects through a linker of nucleoskeleton and cytoskeleton (LINC) complex (402). Current sequence information from genome and transcriptome databases shows that the main proteins from this LINC complex (SUN and KASH) have undergone a remarkable diversification over the course of evolution (403). In particular, concomitant with the increase in complexity of the organism, the number of SUN proteins has significantly increased as well. Lower organisms, such as yeast and cellular slime molds, appear to cope with only one SUN domain protein. Nematodes and flies both contain two genes for SUN proteins, whereas the mammalian genome encodes five distinct members of the SUN protein family: Sun1, Sun2, Sun3, Sun4/SPAG4, and Sun5/SPAG4L (403). Some evolutionary studies have been performed in plants as well (404).

In the mouse, two LINC complexes have been implicated in spermatid elongation: SUN3/Nesprin 1 complex, which connects the manchette to the nucleus, and SUN1/Nesprin 3, which shows an atypical nonnuclear localization at the anterior pole (348). SUN4 has been shown to interact with SUN3/Nesprin 1 complex. SUN4 knockout shows disconnection of the manchette with the nuclear envelope resulting in round-headed sperm (397, 405). Similarly, SUN3-knockout male mice are infertile, displaying a globozoospermia-like phenotype, where nuclei fail to elongate because of the absence of manchette microtubules and perinuclear rings (396). However, *Sun5* mutant mouse spermatozoa contain tailless heads (406). Similarly, biallelic *SUN5* mutations in humans cause male infertility due to autosomal-recessive acephalic spermatozoa syndrome instead of globozoospermia (407). These mutant models have proposed essential roles for the LINC complex in nuclear remodeling; however, the entire interactome of proteins that localize to the manchette and interact with the LINC complex is not well known. Further research dissecting these molecular interactions may bring light to the teratozoospermia defects.

The manchette also participates in the transport of proteins between the nucleus and the cytoplasm. In this context, the motor protein KIFC1 and the nucleoporin protein NUP62 have been shown to interact and participate in this transport mechanism (348). RANBP17 is a RAN-binding protein also involved in transport in and out of the nucleus. It interacts with SPEM1 and UBQLN1 (393, 408). The exact role for SPEM1 is unknown, but its interaction with UBQLN1 may associate this complex with the degradation of unwanted proteins via the ubiquitin-proteasome system.

Proteomic studies of different model organisms have provided very valuable information relevant to our understanding of the functions of the germ cell and conserved key elements. However, to the best of our knowledge, there are no proteomics studies focused on the manchette. Notwithstanding, some proteins that localize to the manchette are conserved during evolution (e.g., ODF1, ODF2, SPAG17, SPAG6) (409, 410). It would be interesting to know whether proteomic studies can find associations between the shape of the sperm head and manchette proteins among different species. In humans, several genes have been associated with teratozoospermia, including *SPATA16*, *DPY19L2*, *PICK1*, *ZPBP1*, and *CCDC62*, that are linked to globozoospermia. Additionally, mutations in *SUN5*, *PMFBP1*, *TSGA10*, *DNAH6*, *BRDT*, and *CEP112* were implicated in acephalic spermatozoa syndrome (411). However, none of the proteins encoded by these genes has been reported to localize to the manchette yet (17). Therefore, it would be important to study the subcellular localization of these proteins in elongating spermatids to dissect whether the manchette is involved in the etiology of human teratozoospermia as it is in the mouse.

Little is known about the role and molecular mechanisms of the manchette in spermatid differentiation in species other than the mouse. Therefore, a comparative overview of common or divergent processes is not possible at this time. Some descriptive studies of the morphology of this organelle exist in some species (35, 350–354, 412), but it is unclear how the manchette relates to nuclei of sperm with other shapes. It may be possible to predict differences between distant species with different nuclear shape, but it should also be borne in mind that there are examples of closely related rodent species in which there is a drastic switch from a falciform nucleus to an oval nucleus in species in which there appears to be a “reversal” to an ancestral head form. The question thus arises as to how this major remodeling of the sperm head is taking place in different species. Furthermore, there is very little information regarding evolution of molecules controlling manchette formation (or participating in manchette function). It is not clear, therefore, what degree of conservation exists in these molecules and whether one or some of these molecules are positively selected. If this were the case, it would be important to learn if this is influenced by postcopulatory sexual selection.

### 5.4. The Nuclear Membrane

The nuclear envelope (NE) is a double membrane around the eukaryotic nucleus. It is structurally composed of two bilayers of lipids and associated proteins similar to the composition of the plasma membrane. These bilayers form the inner and outer nuclear membranes, which are connected by nuclear pore complexes allowing for nucleocytoplasmic trafficking. Lamin A/C and DPY19L2, both NE proteins, have been associated with the attachment of the acrosomal vesicle to the anterior NE margin of spermatids and nuclear shaping (344, 413). Downregulation of Lamin A/C expression in mouse testis leads to severe acrosome and nuclear morphology defects (344). Deletion of *Dpy19l2* in a murine model results in defective spermiogenesis. Round spermatids show fragmented and partially missing nuclear dense lamina. Elongating spermatids show a detached acrosome-acroplaxome complex, dislocation in the manchette structure, and impaired nuclear elongation (413). In humans, deletion of the *DPY19L2* gene leads to globozoospermia, a condition in which spermatozoa show a monomorphic rounded head. Globozoospermic sperm are unable to adhere to or penetrate the zona pellucida, leading to primary infertility (411).

Several B-type lamins and intra-nuclear membrane (INM) proteins are also involved in nuclear shaping via remodeling of the spermatid chromatin. During the initiation of nuclear compaction and reshaping, Lamin B, LAP1, LAP2, and LBR display specific spatiotemporal expression patterns. At the beginning they distribute homogeneously or in one-half of the NE of round spermatids. As spermatid elongation takes place, these proteins translocate to the posterior nuclear pole. This spatial reorganization of proteins is believed to be responsible for chromatin redistribution and replacement of histones by protamines, accounting for changes in the nuclear volume (327).

The NE has been also shown to influence nuclear shape by modulating the flexibility of the nuclear lamina network. In this context, Lamin B3 has been shown to have a dynamic distribution in elongating spermatids. Initially, the protein distributes around the NE and diffusely in the nucleoplasm of round spermatids, occupying one-half of the mouse nucleus. As spermiogenesis progresses, Lamin B3 relocates to the posterior nuclear pole (414, 415) and provides a localized increased flexibility in the nuclear lamina. Interestingly, the distribution of some NE proteins during spermatogenesis is different in germ cells from mouse, rat, and human (327), raising the question about whether these differences may account for their different sperm head shapes.

The nucleus maintains extensive contacts with the cytoskeleton. This is accomplished largely through the LINC complex. LINC complexes are versatile structures recognized in all nucleated cells. They are composed of SUN and KASH domain proteins. SUN proteins localize to the inner nuclear membrane and provide a connection to nuclear structures while acting as a tether for outer nuclear membrane KASH proteins. On the other hand, KASH provides binding sites for diverse cytoskeletal components. In this way, LINC complexes form bridges linking the NE to the cytoskeleton (416). Recent studies identified quite striking polarization in the distribution of the LINC complex during sperm differentiation. SUN3 and Nesprin1 are exclusively found at the posterior NE region linked to the manchette microtubules. However, Sun1η/Nesprin3 and Sun5 (Spag4L) are present at the opposite, anterior spermatid pole, likely linking to the actin cytoskeleton in the acroplaxome (FIGURE 8*C*) (403). Together, these findings suggest that LINC complexes connect the differentiating spermatid nucleus to the surrounding cytoskeletal structures (microtubule manchette and actin filaments in the acroplaxome) to transfer forces that help with nuclear shaping and elongation.

### 5.5. Chromatin Compaction and Protamines

Heretofore, we have considered molecular mechanisms involved in sperm formation that would operate in the interaction of Sertoli cells and the germ cells or that may exist in the germ cells external to the nucleus. Next we consider molecular aspects of nuclear chromatin compaction and the role of protamines.

In contrast to the situation in somatic cells, in which nuclear chromatin is compacted by histones, in sperm cells chromatin compaction is mainly linked to protamine binding to DNA. Replacement of somatic histones by protamines is gradual and actually takes place in three steps. Somatic histones are first replaced in part by testis-specific histones that are amino acid sequence variants of somatic histones. Then, testis-specific histones are replaced by transition nuclear proteins (TNPs). Finally, both testis-specific histones and transition proteins are replaced by highly basic protamines. In each step, replacement is not complete. Thus, a small fraction (1–15%) of histones remain bound to sperm DNA. Sperm nucleosomes are enriched at loci of developmental importance, including imprinted gene clusters, microRNA clusters, and HOX gene clusters (417, 418). However, recent reports claim that sperm nucleosomes remain predominantly within distal gene-poor regions and are depleted in promoters of genes for developmental regulators (419, 420).

The first noticeable change in chromatin structure occurs when the sperm-specific histone H1t variant is deposited in spermatid chromatin, which is transformed into a more uniform and granular state (421). The synthesis and deposition of TNP2 precedes that of TNP1 (422, 423). With the appearance of TNPs, the chromatin starts to condense, with such condensation taking place in an apical to caudal direction (424, 425). When TNP deposition is completed, the chromatin appears to be uniformly condensed. With protamines, the chromatin is reorganized yet again (421).

#### 5.5.1. Role of protamines.

Protamines are nuclear proteins, and they are only expressed in haploid male germ cells (spermatids and the spermatozoon). Protamine genes are expressed in round spermatids soon after completion of meiosis. Transcripts are stored for various days until protein synthesis starts in elongating spermatids (426). Because protamines are important in DNA compaction, modifications in their expression and function have an impact on sperm morphology and performance (427). However, it is not clear whether protamines drive nuclear remodeling by way of nuclear/chromatin compaction, and in turn cell reshaping, or if there is a joint nuclear-cytoplasmic action remodeling the nucleus and, as a consequence, cell reshaping. In the mouse and rat, chromatin condensation by protamines begins around step 12, when some transformation in nuclear shape has already happened. Therefore, chromatin compaction driven by protamines might not be responsible for the initial morphological changes in the head (299), although they may have a role in the main steps of nuclear reshaping. In any case, it should be borne in mind that the sequence of events starting with histonelike proteins, and continuing with transition nuclear proteins, involves chromatin remodeling and condensation, so, rather than thinking solely of protamines, the process of nuclear remodeling should be viewed as underlain by these sequential steps, and thus modifications from within the nucleus may be as important as those driven from outside the nucleus.

Chromatin compaction results in protection of DNA integrity, minimizing damage by various factors. In addition, compaction leads to gene silencing. This process is reversed after fertilization, when protamines are removed and replaced by histones, DNA is repaired within the capacity of the oocyte, and sperm activates embryo development (428). Protamines thus play crucial roles in the series of events ending in fertilization and are also important for development and the well-being of the offspring.

Two types of protamines have been identified in mammals: protamine 1 (PRM1) and protamine 2 (PRM2). The former is expressed in all mammals, whereas the latter only appears to be expressed in primates and most rodents and in a few other species (426). Whereas PRM1 is synthesized as a mature protein, PRM2 is synthesized as a precursor, which is processed as it binds to DNA. As soon as the precursor binds to DNA, it is subjected to proteolytic processing, which finally results in the removal of ∼40% of the NH_2_ terminus (429). Thus, two PRM2 variants are recognized: the precursor form (pre-PRM2) and the processed form (mature-PRM2). The domain that is sequentially cleaved off is collectively termed cleaved-PRM2. As a result of this processing of PRM2, although transcription starts at about the same time as for PRM1, there is a delay in the expression of mature-PRM2 protein because of such processing.

PRM1 is a 50-amino acid nuclear protein with three characteristic domains: a central arginine-rich DNA-binding domain exhibiting 3–11 consecutive arginine residues, which on both sides is flanked by short serine- and threonine-containing segments containing phosphorylation sites. PRM1 in eutherian mammals also has cysteine residues, which can form disulfide bridges between protamine molecules, linking them tightly together. PRM1 is localized within the major groove (430), with one PRM1 molecule being bound per turn of DNA helix, which is equivalent to the binding of 10–11 base pairs of DNA per PRM1 molecule (427). PRM2 shares 50–70% sequence identity with PRM1. Since it is larger than PRM1, it can bind up to 15 base pairs of DNA. The fully processed form of the PRM2 precursor (i.e., mature-PRM2) is slightly larger than PRM1, with 63 amino acids in the mouse (426).

Although it is well known that protamines serve to compact chromatin and prevent DNA damage, it is not yet clear how this happens at the structural-molecular level. Intra- and interprotamine disulfide bonds between cysteines are responsible for the DNA condensation, but it is not known how protamine cross-links with DNA and how compaction takes place through disulfide bonds. The absence of crystallographic data of protamine-DNA complexes has prevented modeling, but some proposals exist for mouse, human, and bull PRM1 (426, 431–433). Additional studies to unravel mechanisms of protamine-DNA interaction are warranted because of the important relation between deficient protamination and the etiology of DNA damage in humans and other species that can result in sub- or infertility or can give rise to genetic defects in offspring (421, 434–437).

#### 5.5.2. Protamines and sperm head shape.

Several lines of evidence suggest that transition nuclear proteins and, above all, protamines are important in reshaping the sperm nucleus during spermiogenesis and thus contribute to the shape of the sperm head. Genetic alterations associate with modifications in sperm head morphology, as seen in studies of disruption of these genes. There is also supporting evidence from correlational studies of sequence evolution and head morphology and from analyses of divergence in expression and sperm head shape. Furthermore, information from humans and domestic species suggests a link between abnormal protamine protein levels and sperm abnormalities.

The relationship between sperm head shape and transition nuclear proteins has been examined after targeted deletion. In mice lacking TNP1, only subtle abnormalities were observed in sperm morphology (438). However, sperm motility was reduced severely, and ∼60% of *Tnp1*-null males were infertile (438). It thus seems that TNP1 may be not essential for histone displacement or chromatin condensation. The absence of TNP1 could be compensated by TNP2, but the dysregulation of nucleoprotein replacement nevertheless leads to a reduction in sperm function and fertility. Spermatogenesis in *Tnp2*-null mice was almost normal (439). There was only a slight increase of sperm retention in stage IX to XI seminiferous tubules, and epididymal sperm showed an increase in abnormal flagella but not in head morphology. *Tnp2*-null mice were fertile but produced small litters, which suggests that TNP2 may not be critical for nuclear shaping or fertility but is necessary for subsequent normal processing of PRM2 and the completion of chromatin condensation (439). The *Tnp1*- and *Tnp2*-null double-mutant mice revealed that nuclear shaping, transcriptional repression, histone displacement, and protamine deposition proceeded relatively normally, but chromatin condensation was irregular in all spermatids. In these mice, epididymal spermatozoa were drastically reduced in number and were highly abnormal, and the mice were sterile. Over 80% of epididymal spermatozoa were dead, and most of the live ones were immotile. Almost all had abnormal head morphology, with many exhibiting blunted apical tips. Most spermatozoa had missing mitochondria or abnormal configurations of the tail, and many were aggregated in clumps. Interestingly, microinjection of testicular or caput epididymal sperm from *Tnp1*^−/−^ *Tnp2*^−/−^ males into intact oocytes resulted in normal embryonic and fetal development and yields of live born equivalent to wild type, whereas, in contrast, cauda epididymal sperm from *Tnp1*^−/−^ *Tnp2*^−/−^ mice produced lower implantation rates and yields of live born than those from wild-type mice (440–442). These results reveal therefore that in *Tnp1*- and *Tnp2*-null mice inadequate sperm formation due to the absence of TNP action during spermiogenesis is exacerbated during epididymal transit, with a major decline in sperm functional capacity.

Disruption of one copy of the *Prm1* or *Prm2* genes in the mouse resulted in a reduction in the amount of the respective protein (443). In these males, there was an alteration in nuclear formation, processing of PRM2, and normal sperm function, suggesting that both protamines are essential (443). There was an increase in morphological abnormalities, and the alterations seen most frequently were sperm with elongated heads having a reduced ventral flexure or sperm heads narrowed and reduced in curvature at the tip. Interestingly, another abnormality frequently observed was sperm with the flagellum tightly wrapped around the head. There were also differences in the degree of chromatin compaction in mature sperm (443). Further studies focusing on PRM2-deficient sperm confirmed the occurrence of head abnormalities and a less pronounced ventral flexure than that seen in wild-type mice. In some cases, the PRM2-deficient sperm had the head folded back onto the flagellum (444). In addition, these studies found, as expected, alterations in the organization and integrity of sperm DNA and, at the ultrastructural level, reduced compaction of the chromatin. Microinjection of spermatozoa that were PRM2 deficient resulted in activation of most oocytes, although few oocytes reached the blastocyst stage, suggesting damage to the paternal DNA (444). In another study (445), heterozygous *Prm1*^+/−^ male mice produced with embryonic stem (ES) cells revealed that >70% of spermatozoa were abnormal. Most abnormalities (40%) were seen in the sperm flagellum, ∼20% of sperm had abnormal heads, and ∼10% of sperm had abnormalities in both. A proportion of these sperm were capable of fertilizing zona-free oocytes in vitro and to generate offspring despite having abnormal head shapes, destabilized DNA, and other abnormalities (445). In a third study, using CRISPR/Cas9-mediated gene editing in oocytes, a *Prm2*-deficient mouse was established (446). In contrast to a previous report (443), heterozygous males were fertile, with sperm displaying normal head morphology and motility. On the other hand, *Prm2*-deficient sperm showed impairment of DNA condensation and sperm acrosome formation. Sperm counts of Prm2^−^/^−^ males were normal, and so were testis weight and architecture in relation to control animals, but males were sterile (446). Spermatozoa displayed severe membrane defects that resulted in lack of motility (446).

One important conclusion from work in which transition nuclear protein or protamine genes have been disrupted is that, in addition to effects on the morphology of sperm nucleus, which these proteins remodel, there are also significant effects on other sperm structures such as the flagellum. This suggests a concerted action during sperm formation, even for proteins acting in the nucleus.

#### 5.5.3. Protamine evolution and sperm head shape.

Studies on sequence evolution suggest that PRM1 may have evolved from an H1-like histone (447). Protamine genes have gradually increased in length during vertebrate evolution. There is high heterogeneity between different species reflecting a rapid rate of *Prm1* gene evolution (448). At gene level, variability in protamine is higher in exons than in its intron (449), whereas the 3′-end seems to be the most variable region (450). At the level of the protein, the relative proportion of arginine residues is rather constant (50–70%) but the position of arginine residues is highly variable (427). The conservation of a high arginine content may be driven by the function of protamine in sperm chromatin condensation, because protamines with a higher arginine content can form DNA complexes that are more stable. The analyses of PRM1 evolution in eutherian mammals have suggested that arginine content of this protamine is negatively correlated with head width (451), thus resulting in a more compact or elongated head, potentially contributing to higher swimming efficiency.

The *Prm2* gene seems to have duplicated from the *Prm1* gene (426), but the question regarding the origin of PRM2 still remains unanswered. Comparative studies in rodents have revealed that cleaved-PRM2 is likely to play a role in producing small and elongated heads, making sperm more competitive, whereas there is no association between sperm head phenotype and mature-PRM2 (452, 453). Divergence in *Prm2* promoter, presumably having an effect on gene regulation, was associated with differences in swimming speed, probably through an effect on head shape (454). Rats have reduced PRM2 levels that appear to be due to suppression at both the transcriptional and translational levels (455). Some hamster species (common European hamster, Chinese hamster) entirely lack PRM2 (456, 457).

All primates express PRM2, but differences exist in the level of expression, and this may relate to chromatin remodeling and nuclear shape. In humans, apes, and Old and New World monkeys, the processed forms of PRM2 contain 57 amino acids (458). Ruminants (e.g., bull, ram, and goat) and boar have a *Prm2* gene but appear to lack a functional PRM2 protein or show very low levels of expression (426, 427). The absence of PRM2 in these species seems to be caused by point mutations that result in a loss of arginine residues representing the DNA-binding domain and an accumulation of hydrophobic amino acids, both of which adversely impact the binding affinity of PRM2 to DNA (459). Sperm of perissodactyls (horse, zebra), lagomorphs, and elephants have two PRM2 variants (pre-PRM2 and mature-PRM2) (426). Information on PRM2 protein in sperm of other species is scarce. No species has been reported that only expresses PRM2, and, as a matter of fact, the content of PRM2 does not seem to exceed 80% in any species (427). This would indicate that the presence of PRM2 alone is not enough for proper DNA condensation.

The considerable variation existing in the proportion of PRM1 and PRM2 proteins in different species may relate to the possibility that the two protamines interact with each other and bind to DNA in a consistent manner. Therefore, the predominant protamine function seems to relate to the neutralization of the negative charges along the DNA phosphodiester backbone and, as a result, to enable adjacent molecules of DNA to be packed close together. The process of compaction continues during the period of transit along the epididymis, as seen in the mouse (460), during which time the protamines form intramolecular (PRM1) and intermolecular (PRM1 and PRM2) disulfide bridges between the cysteine residues (433), resulting in the sperm DNA being condensed to a volume that is one-twentieth of the volume of a somatic nucleus.

The proportion between PRM1 and PRM2 (the protamine ratio) seems to be tightly regulated within a species and varies considerably between species, in contrast to the total protamine to DNA mass in sperm nuclei, which is highly similar between different species (427). Variation in the proportion between PRM1 and PRM2 between rodent species is mainly due to variations in PRM2 content (457), and in these species there is much variation in the protamine ratio, even among closely related species (461). In stallions the proportion of PRM1 to PRM2 is ∼3 to 1 (427), and a significantly decreased *Prm1*-to-*Prm2* mRNA ratio associates with low fertility (462). In sperm of men there are approximately equal amounts of both protamines, with aberrant protamine ratios (mRNA or protein) associating with elevated DNA fragmentation and male subfertility (20, 436). An overall comparison of species with only *Prm1* expression versus those with *Prm1* and *Prm2* expression suggests that the latter group has a significantly enhanced likelihood of sperm DNA fragmentation (463).

**FIGURE 9.**
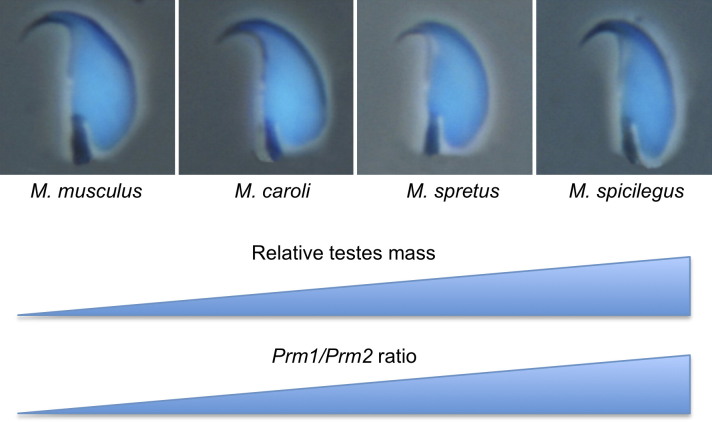
Protamine expression in mouse species and association with sperm head shape. Ratios of *Prm1*-to-*Prm2* mRNA levels vary among closely related mouse species of the genus *Mus* with a parallel increase in sperm competition levels (as revealed by the proxy of relative testis mass). Based on data in Lüke et al. (452).

The shape of the sperm nucleus may be influenced by variations in the protamine ratio, since the latter may influence chromatin compaction. A study in mouse species showed that the variation seen in protamine ratios between species associates with diversity in sperm head shape (461) (FIGURE 9). In the horse, the proportion of morphologically abnormal sperm correlated negatively with the protamine mRNA ratio (which in this study was calculated as *PRM2/PRM1* in contrast to the usual *PRM1/PRM2*), indicating that spermatozoa carrying head defects display a diminished protamine ratio (462). In the bull, abnormal spermatozoa showed either a lack of PRM1 or scattered localization in the apical/acrosomal region of the nuclei (464). In human patients, alterations in protamine ratios are usually associated with abnormal spermatozoa. Deviations of PRM1/PRM2 above or below average values are associated with infertility, and patients with altered PRM1/PRM2 are more likely to display a higher frequency of abnormal morphology, along with decreased sperm concentration and motility, and reduced penetration capacity, compared with subjects with a normal protamine ratio (465).

Because of the role of protamines in nuclear condensation and the overall regulation of sperm head formation, these proteins have a major impact in the structure of the entire sperm cell and, by extension, in its function. Because differentiation of the sperm cell during spermiogenesis is also a concerted process, when both reorganization of the nucleus and the entire head and formation of the flagellum take place, concomitant alterations in head and flagellum do occur, in such a way that defects in protamine synthesis result in sperm with no or reduced motility. Moreover, defects in the cytoplasm-nuclear transport via the manchette can also lead to altered nuclear translocation of protamines. Some studies have evaluated the localization of protamines during spermatogenesis (reviewed in Ref. 466). Subcellular localization of protamines was also studied in isolated mouse (467–469) and human (470) spermatids. However, this is an area that needs further mechanistic studies. For instance, it is not known how protamines are transported from the cytoplasm to the nucleus. Additional relevant questions are: What is the protein that recruits protamines to the intramanchette transport? How is the complex protamine-recruiter-transporter targeted and delivered to the nucleus? Are protamines transported via vesicle or nonvesicle transport? It has been shown that *Prm1* and *Prm2* are translated at the same time (427), but are both protamines transported to the nucleus also at the same time? If there is a differential transport of PRM1 and PRM2, is this associated with PRM1-to-PRM2 ratios at different steps in spermiogenesis and this in turn with different nuclear shapes in different species? Because protamines have an important role in chromatin compaction, protection of DNA, and epigenetic marks, and this translates into how the sperm nucleus condenses and the sperm cell acquires its final shape, understanding the links between these various molecular and cellular processes is important for a thorough knowledge of determinants of fertility.

#### 5.5.4. Heterologous expression of protamines and nuclear shape.

Studies designed to examine cell reprogramming for cloning by means of somatic cell nuclear transplantation afforded the unexpected opportunity to analyze nuclear remodeling following protamine transfection. The rationale of this work was that, similarly to the reprogramming during differentiation of the male germ line, somatic cell nuclei could be reprogrammed to enhance the efficiency of cloning if submitted to a similar process under the influence of protamines. It was found that human PRM1 induces compaction of sheep fibroblast nuclei and gene silencing (471). The shape of these nuclei resembled those of highly compacted nuclei of spermatozoa. When such nuclei are microinjected into enucleated sheep oocytes, they undergo efficient protamine-to-maternal histone exchange and develop into normal blastocysts, demonstrating the functionality of such nuclei (471, 472). Moreover, embryos thus generated develop to the blastocyst stage with a quality similar to embryos derived from in vitro fertilization (IVF), thus underscoring the physiological competence of protaminized somatic cell nuclei. Subsequent work showed that mouse PRM1 can also remodel and condense fibroblast nuclei and that PRM1 from both human and mouse can induce compaction of either sheep or mouse fibroblast nuclei (472). Additional work revealed that efficiency of nuclear remodeling could be enhanced by inducing nuclear quiescence and histone hyperacetylation before transfection of fibroblasts (473). Such pretreatment promoted a much higher conversion of somatic nuclei into spermatid-like structures. Future work will likely benefit from using this system and will allow exploration of mechanisms compacting nuclei in a way that resembles that achieved in sperm. Furthermore, this approach would allow us to ask whether nuclei may be remodeled and shaped by intrinsic factors in the nucleus and its dependence on protamine sequence and expression. Thus, although this system represents a simplification of mechanisms underlying nuclear remodeling during spermatogenesis, it could be an outstanding method to examine DNA reorganization to infer processes taking place during gamete formation.

## 6. CONCLUSIONS AND FUTURE DIRECTIONS

This review has attempted to integrate recent important findings regarding sperm bauplan and function, discussing aspects of sperm morphology (structure, shape, size) and performance in an evolutionary perspective and highlighting areas that deserve further investigation. There is a rich variety of sperm head shapes among vertebrates. Globular forms are common among fish, but there are also filiform or helical sperm heads. Amphibia have filiform, cylindrical, tapering, or long heads. Birds’ sperm heads are filiform or helical. Flattened ovoid heads are predominant in mammals, although murine and cricetid rodents generally have a falciform, sickle-shaped head. There is also considerable diversity in flagellum structure and dimensions. Changes in sperm shape and size influence sperm swimming patterns, but there is still a paucity of information on sperm biomechanics or hydrodynamic efficiency. Furthermore, considering that spermatozoa perform in aqueous environments or relatively viscous fluids in the female tract, such factors should be considered in attempts to understand sperm behavior. In addition to a general positive association between sperm shape or dimensions and swimming velocity, processes of ATP generation and consumption may also have an important bearing on sperm survival and performance. Unfortunately, there is still very limited knowledge of pathways for, and regulation of, generation of sperm ATP, with considerable differences probably existing between species. It is also worth considering that sperm may change environment as they approach the oocyte, and that the sperm pattern of movement changes as they reach the site of fertilization. These changes may influence the sperm’s bioenergetics, particularly ATP consumption. The interactions between sperm cells and female- or oocyte-derived factors thus require continued attention.

Diversity of sperm morphology and performance could be examined in an evolutionary framework. In this context, the inter- and intraspecies diversity, and also intramale variation, could be analyzed to understand evolutionary history of sperm cells. Issues such as direction of trait evolution, origin of biological novelties (e.g., the appearance of head asymmetries or head appendices), sperm developmental constraints, and reversal of complex traits (such as the reappearance of simple heads in several rodent lineages) represent important questions that would command considerable attention in the future. Sperm cells may thus be good models to address these important biological phenomena. Sperm morphology and function have likely evolved under the influence of various selective forces, namely the mode of fertilization and postcopulatory sexual selection, in the framework of common descent. Vertebrates differ in their mode of fertilization, and therefore sperm and oocytes vary in the way they meet and interact. In externally fertilizing species, sperm structure is rather simple. Internal fertilization led to a series of modifications resulting in complex and longer spermatozoa with modifications in their bioenergetics and swimming behavior. In internal fertilizers, sperm cells have to swim actively, at least during part of their journey, to reach the site of fertilization. The distance to swim may vary from a few millimeters in small species to a meter or more in some large species; variation also exists in the time sperm need to survive in the female tract until the moment of fertilization. Therefore, sperm traits have likely evolved in response to both spatial and temporal determinants. Additional changes in sperm morphology or performance may relate to the interaction between sperm and oocytes that may vary in cellular or acellular coats. In internally fertilizing species, reproductive mode (oviparity vs. viviparity) may impact on characteristics of the female tract or the interval between sperm deposition and fertilization, which, in turn, may influence spermatozoa. Some progress has been made, by using comparative studies, in our understanding of the role of mode of fertilization on sperm evolution, but there are still many questions left unanswered with regard to interactions between sperm cells and the female tract and the sperm-egg interaction.

Processes of sexual selection occurring after copulation may relate to sperm selection in the female tract (i.e., cryptic female choice) or competition between ejaculates to achieve fertilization (i.e., sperm competition). In the former, sperm cells of certain males may be differentially favored on the basis of genotype or phenotype. This could involve influences on sperm swimming performance via reproductive fluids, including processes of chemoattraction, or may relate to choice during oocyte-sperm interaction, including processes of ovum defensiveness. In the latter, sperm will compete in the female tract when females mate with two or more males during their period of sexual receptivity; in external fertilizers, it would happen when massive concomitant spawning takes place. For these selective processes, the evolutionary framework is rather well understood and theory and modeling have received considerable attention. On the other hand, much needs to be done to understand their underlying cellular and molecular mechanisms. Many comparative studies have focused on how these selective forces could influence sperm traits. The major effect of sperm competition on sperm numbers is now clear, but the impact on sperm morphology and performance needs additional attention. There seems to be an evident, positive, overall effect of sperm competition on whole sperm size, and proportions of different compartments; less has been explored with regard to shape. Information on influence on bioenergetics is now beginning to emerge. There seems to be a complex interaction between morphology (shape, size), bioenergetics, and swimming, so multifactorial analysis of trait evolution under sexual selection is needed. A more detailed dissection and examination of sperm kinematic parameters after sperm activation or hyperactivation and characterization of sperm subpopulations would be most helpful in the future. Functions such as capacitation and the acrosome reaction in response to natural ligands, and the underlying cellular and molecular mechanisms, need to be examined under the light of evolutionary forces. How these selective forces operate with regard to both sperm production (e.g., testis size and architecture or kinetics of sperm formation) and differentiation and cell shaping and remodeling need to be analyzed to understand the processes that prepare sperm to perform (in an external environment or in the female tract) and to acquire the competence to reach and interact with the oocyte at fertilization.

Sperm formation is under a strict genetic control, which comes into play during the process of spermatogenesis in the testis and subsequent maturation after its release. Importantly, sperm development is also under the influence of environmental factors. There are still many unknowns regarding the regulation of mechanisms driving spermatogenesis. The question arises as to the possible rate of evolution of genes involved in proliferation versus differentiation, considering that the former may be more conserved than the latter, as suggested by experiments with spermatogonia transplantation. Moreover, it will be important to identify molecules essential for sperm formation that could be the subject of comparative-evolutionary studies. For example, proteins like SPAG17, ODF1, ODF2, and dynein were found to be positively selected during evolution and are also essential during spermatogenesis. Whether these proteins interact as a complex or in similar mechanisms is unknown.

**FIGURE 10.**
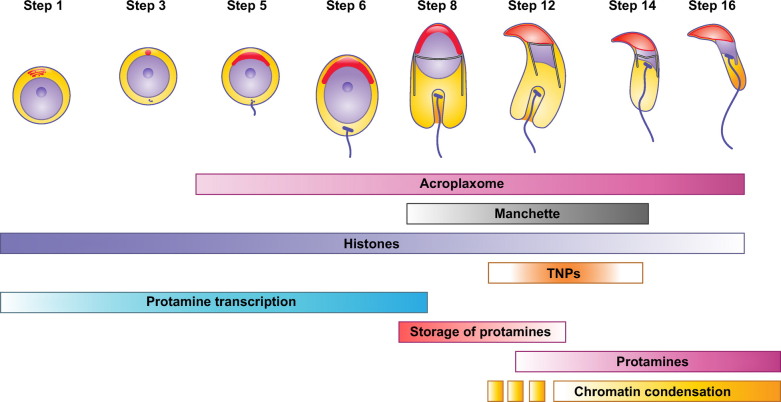
Schematic representation of the internal mechanisms supporting the remodeling of the spermatid head. In early spermiogenesis steps the F-actin filaments and associated proteins forming the acroplaxome may be responsible for the first changes in nuclear shaping followed by the forces generated by the microtubules and actin filaments in the manchette and the interaction with the nuclear envelope through the linker of nucleoskeleton and cytoskeleton (LINC) complex, and final twists may be supported by chromatin condensation due to replacement of histones by transition nuclear proteins (TNPs) and then protamines.

Several mechanisms may be responsible for nuclear shaping. Depending on the species, some may be more important than others. In mammals, particularly in rodents, the cytoskeleton structures seem to play crucial and coordinated roles. For instance, in early spermiogenesis steps (around step 5) the F-actin filaments and associated proteins forming the acroplaxome may be responsible for the first changes in nuclear shaping. This may be followed by forces generated by the microtubules and actin filaments in the manchette and the interaction with the NE through the LINC complex. Final twists could be supported by chromatin condensation due to replacement of histones by transition nuclear proteins and then protamines (FIGURE 10). In any case, the possibility that chromatin compaction promoted by protamines could have a more relevant role in head shaping should not be overlooked.

Regarding regulation of chromatin condensation and how this leads to a sperm cell that is transcriptionally silent, and how epigenetic information is transferred from the sperm to the oocyte, there are still many gaps in knowledge. Questions that remain to be answered relate to the evolution of sperm protamines, their expression and transport during the final stages of sperm cell differentiation, the impact of protamine-mediated DNA compaction on sperm structure and function, and, in an overall integrative fashion, examination of the interaction between different factors that influence sperm formation and function.

Characterizing and understanding the fundamental aspects of the sperm bauplan, in connection to both evolution and function, would lead to a better understanding of fertility and would allow for accurate diagnostic tests and better prognosis and treatment, and also reliable predictive assessments of both animal and human fertility. Furthermore, it would contribute to learning about the impact of environmental factors and lifestyle on the integrity of DNA and effects on future generations.

## GRANTS

This work was supported by the National Institutes of Health (Grant R03 HD-101762), the Spanish Ministry of Science and Innovation (Grants CGL2016-80577-P and PID2019-108649GB-I00), and Consejo Superior de Investigaciones Científicas (Grant 2019AEP165).

## DISCLOSURES

No conflicts of interest, financial or otherwise, are declared by the authors.

## AUTHOR CONTRIBUTIONS

M.E.T. and E.R.R.S drafted manuscript; prepared figures; edited and revised manuscript; and approved final version of manuscript.
